# Variants in human *CD48* lead to impaired T cell immunity and increased inflammation

**DOI:** 10.1172/JCI191340

**Published:** 2026-04-14

**Authors:** Samantha Milanesi, Tiziana Lorenzini, Tommaso Marchetti, Diana Tintor, Raquel Planas, Ola Sabet, Lars Malmström, Sudip Acharya, Carson D. Williams, Zoe E. Manning, Jack H. Roser, Angelica C. Ehler, Michael Huber, Seraina Prader, Stefano Vavassori, Cullen M. Dutmer, Jordan K. Abbott, Jana Pachlopnik Schmid

**Affiliations:** 1 Pediatric Immunology, University of Zurich, and the Children’s Research Center, University Children’s Hospital Zurich, Zurich, Switzerland.; 2 Department of Cell Biology, Physiology and Immunology, University of Barcelona, Barcelona, Spain.; 3 Children’s Cancer Hospital Egypt 57357, Cairo, Egypt.; 4 University of Colorado School of Medicine, Department of Pediatrics, Section of Allergy and Immunology, Aurora, Colorado, USA.; 5 Institute of Medical Virology, University of Zurich, Zurich, Switzerland.; 6 Division of Pediatric Immunology, University Children’s Hospital Zurich, Zurich, Switzerland.

**Keywords:** Immunology, Inflammation, Cellular immune response, Genetic diseases, T cells

## Abstract

CD48 is a surface molecule with immunoregulatory functions. Following our initial report of a patient with a de novo heterozygous variant at amino acid S220 in the *CD48* gene, we describe a second, unrelated patient with similar features of immune dysregulation and a missense change affecting the same residue. To further elucidate the specific pathogenic mechanisms of the identified variants, we reviewed patient records, analyzed patient-derived cells, and employed complementary in vitro and in vivo model systems, including transfected cell lines and CD48-deficient mice. We demonstrate that the variants are associated with altered distribution of CD48, characterized by diminished CD48 surface expression, intracellular retention, and activation of ER stress signaling. Patient T cells displayed increased susceptibility to apoptosis, reduced antiviral responses, and enhanced inflammation. Both patients exhibited T cell lymphopenia, a restricted T cell receptor repertoire diversity, and oligoclonal expansions consistent with antigen-driven selection. In parallel, virally infected CD48-deficient mice recapitulate key aspects of the human phenotype, including delayed antiviral immune responses, impaired viral clearance, and pronounced inflammation. We conclude that identified variants compromise CD48 cell surface localization, impair T cell survival and function, and predispose to inflammation, thereby highlighting the role of CD48 in immune regulation and the prevention of excessive inflammation.

## Abstract 2

An inborn error in the immunoregulatory molecule CD48 reveals its importance in regulating T-cell responses during infections and preventing excessive inflammation in both humans and mice.

## Graphical abstract

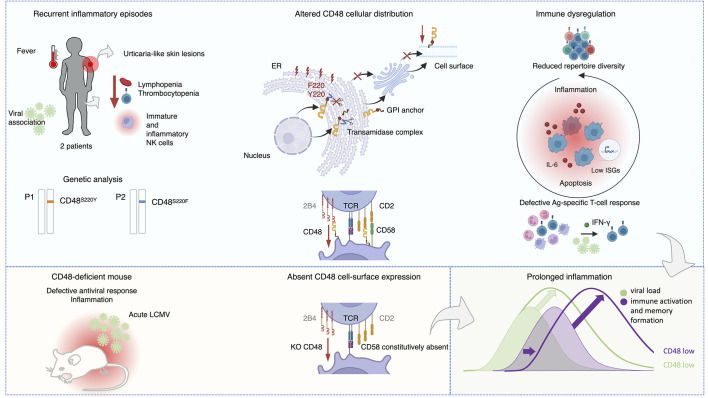

## Introduction

In humans, lymphocyte-mediated cytotoxicity plays a central role in maintaining a balance between pathogen defense and inflammatory control (1, 2). Cytotoxic lymphocytes, such as CD8^+^ T cells and NK cells, eliminate virus-infected cells and APCs by releasing perforin and granzymes from cytotoxic granules. Complete loss of cytotoxicity (due to the lack of perforin or impaired granule release) hinders the clearance of infected cells and APCs and results in prolonged CD8^+^ T cell stimulation. This drives a feed-forward inflammatory loop causing the development of one of the most severe inflammatory reactions known: hemophagocytic lymphohistiocytosis (HLH) (2). Diminished cytotoxicity, as a consequence of insufficient T cell immunity, similarly leads to reduced pathogen clearance followed by heightened immune activation and cytokine release (3). T cell responses are tightly controlled by costimulatory and coinhibitory signals, including those mediated by CD48.

The costimulatory molecule CD48, also known as signaling lymphocytic activation molecule-2 (SLAMF2), is a glycosyl-phosphatidylinositol (GPI)-anchored protein that is widely expressed on leukocytes (4). Both human and mouse CD48 bind CD2 with low affinity (*K_D_* ~100 and ~90 μM, respectively) and 2B4 (CD244, SLAMF4) with moderate affinity (*K_D_* ~8 and ~16 μM, respectively) (4). While CD2–CD48 interactions are required for T cell receptor (TCR) signaling and T cell activation in mice, in humans, similar functions are mediated by CD2–CD58 interactions, as CD2 binds CD58 with high affinity (*K_D_* ~9–22 μM) and CD58 is absent in mice (4, 5). In humans, 2B4 (CD244, SLAMF4), rather than CD2, is the primary moderate-affinity partner of CD48 (4, 6).

Extensive studies of 2B4 signaling — particularly in EBV-triggered HLH and lymphoma in patients with defective intracellular signaling due to SLAM-associated protein deficiency (7, 8) — have demonstrated that the CD48-2B4 interaction can exert context-dependent activating or inhibitory effects on T and NK cell functions (4). Studies of 2B4 blockade in mouse models with humanized immune systems have demonstrated that CD8^+^ T cells — and not NK cells, which require both the CD48/2B4 signal and the absence of Human Leukocyte Antigen class I expression to mediate a cytolytic effect (9) — are the prime effectors in EBV viral clearance and tumor prevention (10). This observation emphasizes 2B4’s key role in modulating CD8^+^ T cell function, probably through interaction with CD48, although the latter was not examined directly in these studies. Human CD48 has mainly been studied in vitro (human cell based). CD48 blockade in vitro has been shown to reduce CD8^+^ T cell cytolytic activity, particularly in T cells expressing low-avidity TCRs (11), while also enhancing TCR signaling and activation in mucosal-associated invariant T cells (12). Furthermore, CD48 has been shown to act as a receptor that senses and binds *Candida albicans*, thereby contributing to host defense, and blockade of this interaction reduced human eosinophil activation (13). These results illustrate the diverse roles of CD48 and its ligands across different cell types, many of which remain to be fully elucidated.

Human *CD48* S220 variant syndrome is a very rare genetic immune disorder (14). Our initial studies of 1 patient with a heterozygous *CD48* S220 variant revealed low CD48 surface expression, reduced soluble CD48 in the blood, impaired maturation and a proinflammatory profile of NK cells, lower susceptibility of target cells to NK cell cytotoxicity, and high systemic cytokine levels (14). Even though this patient demonstrated normal T cell cytotoxicity in standard assays (14), we hypothesized that a T cell dysfunction could secondarily dampen effective CD8^+^ T cell responses, impair viral clearance, and skew the immune response toward inflammatory pathology. In addition, the importance of the S220 residue for correct GPI anchoring prompted us to investigate how S220 *CD48* variants specifically compromise CD48-mediated immune signaling.

Here, we expand on our initial findings by (a) describing the clinical and immunologic characteristics of 2 patients with distinct de novo *CD48* S220 variants; (b) investigating variant-specific effects on T cell homeostasis, proliferation, and TCR repertoire diversity; and (c) evaluating the role of CD48-mediated T cell immunity in promoting efficient antigen clearance and regulating inflammation. By combining insights from human observations with experiments conducted in mutant cell lines and a CD48-knockout (CD48^–/–^) mouse model, we gain a deeper understanding of the role of CD48 in coordinating T cell responses and restraining inflammation during immune challenges.

## Results

### S220 CD48 variants in 2 patients with recurrent, inflammatory episodes.

**Figure 1:**
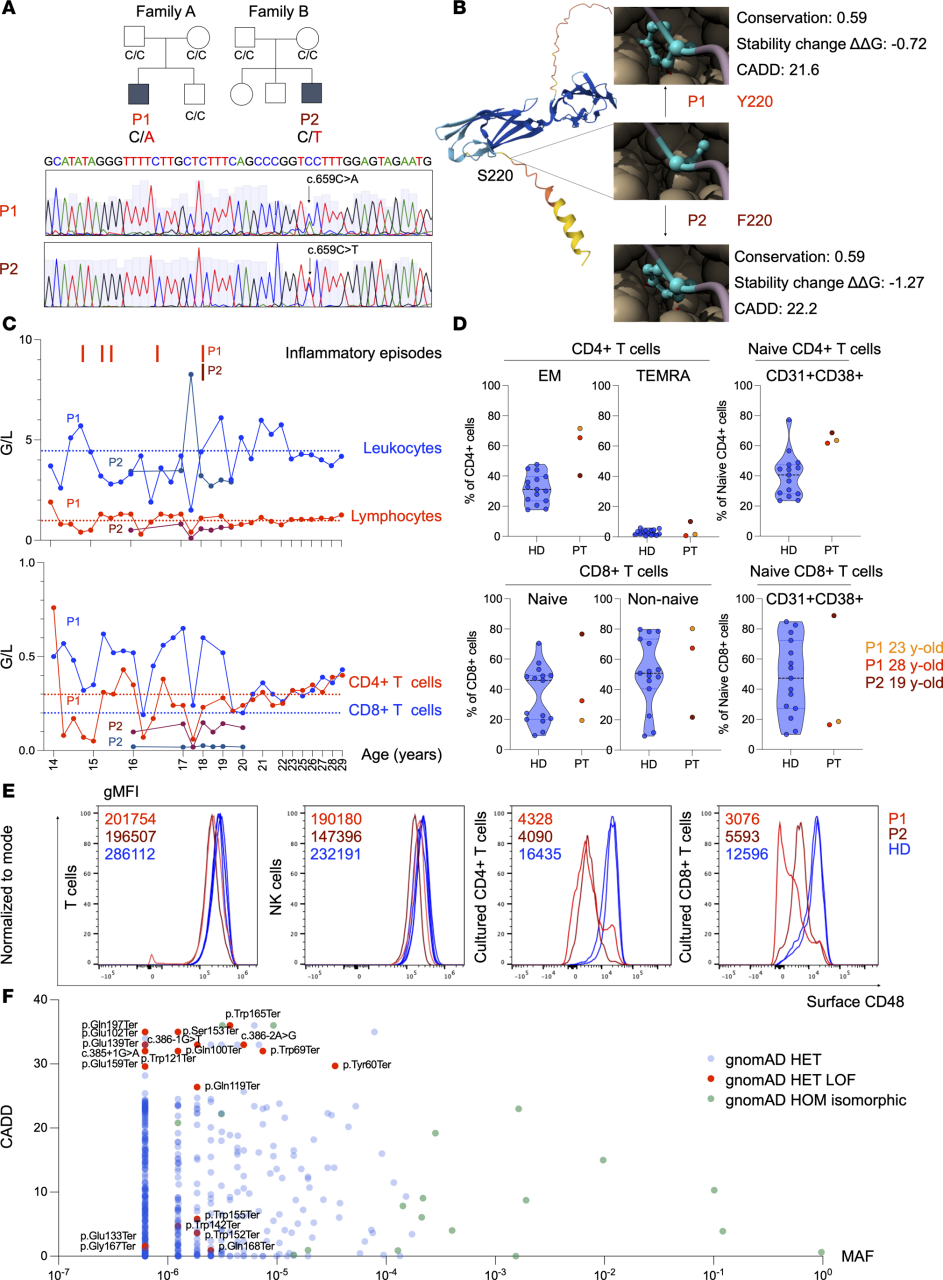
Genetic and immunologic characteristics of patients with different *CD48* S220 variants. (**A**) Family trees showing male (square) and female (circle) family members; open symbols represent HDs, and solid symbols represent the patients (P1 and P2). Below, Sanger sequencing of the *CD48* gene (exon 4), showing the identified variants in both patients. (**B**) The CD48 protein structure predicted using AlphaFold 2 (47) and colored by per-residue confidence (predicted local distance difference test). Blue, very high; light blue, high; yellow, low; orange, very low. The identified ω-site amino acid substitutions were modeled using PDB 8IMY, and the PIGK–UL16-Binding Protein 2 complex was visualized using ChimeraX (48). Conservation, ΔΔG stability change, and CADD scores were obtained using ProtVar (49). Conservation 0.45–0.60, moderate; ΔΔG < 2 kcal/mol, unlikely destabilizing; CADD 20–24.9, likely deleterious. (**C**) Change over time in the leukocyte (blue), lymphocyte (red), CD4^+^ T cell (red), and CD8^+^ T cell (blue) blood counts measured in giga per liter (G/L) in P1 (light red and light blue) and P2 (dark red and dark blue). Red bars indicate inflammatory episodes; dotted lines indicate reference intervals. (**D**) Truncated violin plots showing the median and quartiles of the frequencies of EM, terminally differentiated effector memory (TEMRA), and CD31^+^CD38^+^ naive CD4^+^ T cells, and naive, non-naive, and CD31^+^CD38^+^ naive CD8^+^ T cells in 15 adult HDs (blue dots) compared with P1 (orange and red dots) and P2 (dark red dots). The ages at measurement for P1 and P2 are indicated. Due to the low patient sample size, no statistical analysis was performed. (**E**) Overlaid flow cytometry histograms of CD48 surface expression, reported as geometric MFI (gMFI), in peripheral blood cell subsets in 2 adult HDs (blue), P1 (red), and P2 (dark red). (**F**) CADD–minor allele frequency (MAF) graph of *CD48* variants reported in gnomAD. Heterozygous (HET) variants, light blue; HET loss of function (LOF), red; homozygous (HOM) isomorphic, green.

We investigated 2 male patients of European ancestry from unrelated, nonconsanguineous families with a history of recurrent, inflammatory episodes (Figure 1A). Both patients exhibit heterozygous missense variants in the *CD48* gene, detected by whole-exome sequencing (WES): patient 1 (P1) carries c.659C>A (p.S220Y), as described previously (14), while patient 2 (P2) carries c.659C>T (p.S220F) (Figure 1A). The variants were confirmed to be de novo by Sanger sequencing of family members (Figure 1A). They affect the same residue (the serine at position 220 [S220]) but result in different amino acid substitutions: tyrosine in P1 and phenylalanine in P2 (Figure 1B). Other WES-identified variants were classified as likely benign or of uncertain significance according to the guidelines of the American College of Medical Genetics and Genomics and the Association for Molecular Pathology (15) and were therefore not regarded as plausible contributors to the patients’ phenotypes (Supplemental Table 1; supplemental material available online with this article; https://doi.org/10.1172/JCI191340DS1).

Both patients (at the time of publication, 30 and 22 years old) experienced early-onset inflammatory episodes accompanied by fever and urticarial rashes, which tended to become less frequent with age (Supplemental Figure 1A). Some episodes required hospitalization and were associated with lymphopenia (Figure 1C) and thrombocytopenia. In P1, some (but not all) inflammatory episodes met 5 of the 7 updated HLH-2004 diagnostic criteria (16, 17). An infectious trigger was identified on several occasions, including *Streptococcus pneumoniae* and Varicella-zoster virus (VZV) in P1 and norovirus, rotavirus, and Influenza A (2009-H1N1) in P2 (Supplemental Figure 1A). P1 had CD4^+^ lymphopenia, which was more severe during the inflammatory episodes, but tended to recover with age. P2 had CD4^+^ and CD8^+^ lymphopenia, which persisted at the most recent measurement (Figure 1C). Both patients exhibited a memory CD4^+^ phenotype, with the naive subset showing a relative enrichment of CD38^+^CD31^+^ cells, a phenotype previously associated with recent thymic emigrant–like features (18) (Figure 1D). By contrast, a memory CD8^+^ T cell phenotype was observed only in P1, whereas a relative enrichment of CD38^+^CD31^+^ naive CD8^+^ cells was detected only in P2, consistent with his CD8^+^ T cell lymphopenia (Figure 1D). Moreover, effector memory (EM) T cells in P1 showed a tendency toward a CD57^+^CD27^–^ phenotype, whereas those in P2 tended to express CD38; both patterns have been associated with immune activation (19, 20) (Supplemental Figure 1B). Flow cytometry revealed low levels of CD48 protein surface expression in various leukocyte subsets, with a trend toward further reduction after 4 days of in vitro culture, in both patients (Figure 1E and Supplemental Figure 1C).

We examined public genome databases to identify additional rare *CD48* variants consistent with haploinsufficiency and detected 13 heterozygous rare loss-of-function *CD48* variants with a Combined Annotation Dependent Depletion (CADD) score above 20 in gnomAD v4.1.0 (21) (Figure 1F). Notably, no other S220 *CD48* variants are listed in the database, apart from a synonymous p.S220S variant (21). Collectively, these findings support the presence of additional molecular mechanisms beyond haploinsufficiency that contribute to the observed phenotype.

### Human S220 CD48 variants lead to altered CD48 cellular distribution.

In the ER, GPI anchors are synthesized and attached to proteins that contain a C-terminal GPI attachment signal sequence, such as CD48, after cleavage of the peptide bond at the ω-site by the transamidase complex (22). The S220 residue is the ω-site of the CD48 precursor protein (23). Given that amino acid restrictions are very severe at the ω-site (24), the substitution of a small polar residue (e.g., serine) with a larger amino acid (e.g., tyrosine or phenylalanine) at the GPI linker position is likely to impair cleavage and GPI anchoring, as observed for other GPI-anchored proteins (24). It has been shown that uncleaved precursors of GPI-anchored proteins are retained in the ER and degraded (25).

**Figure 2:**
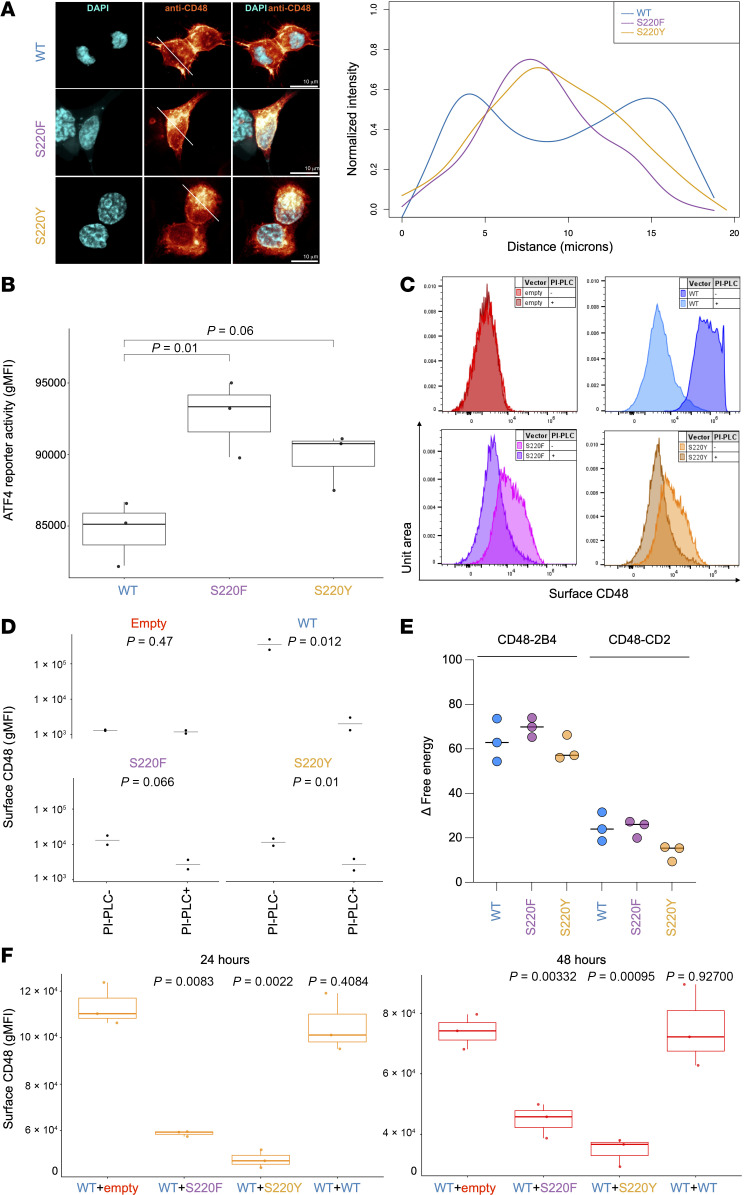
Human S220 *CD48* variants lead to altered CD48 cellular distribution. (**A**) Microscopy analysis and quantification of the CD48 fluorescence intensity in HEK293 cells transfected with a plasmid encoding *CD48* WT (light blue), S220F (purple), or S220Y (orange). Scale bars: 10 μm. (**B**) Box-and-whisker plots showing the median and quartiles of ATF4 reporter activity in HEK293 cells cotransfected with ATF4-mScarlet and either WT, S220F, or S220Y CD48 expression constructs. mScarlet fluorescence was measured by flow cytometry 24 hours after transfection. Expression levels are reported as the gMFI of the mScarlet signal. *P* values were determined using 1-way ANOVA followed by Dunnett’s post hoc test, with the WT used as reference. (**C** and **D**) Flow cytometry quantification of surface CD48 expression in HEK293 cells transfected with an empty vector (red), WT (blue), S220F (purple), or S220Y (orange) CD48 expression constructs, and either left untreated (–) or treated with PI-PLC (+). A representative experiment (**C**) and the relative quantification of the CD48 gMFI (**D**) are shown. The experiment was conducted twice. *P* values were determined by 2-tailed *t* tests. (**E**) Scatterplot of estimated binding free energies of the interaction between CD48 WT (light blue), CD48 S220F (purple), and CD48 S220Y (orange) with 2B4 and CD2. For each sample, the median of 3 measurements is shown. For each ligand, free energies were compared using Friedman’s test followed by Dunn’s multiple-comparison test. No significant differences were detected. (**F**) Flow cytometry quantification of surface CD48 expression in HEK293 cells cotransfected with equal quantities of CD48 WT and either an empty vector, or S220F, S220Y, or WT expression vectors, at 24 hours (left panel) and 48 hours (right panel) after transfection. Statistical comparisons between each experimental group and the control group (WT + empty vector) were performed using separate 2-tailed *t* tests. *P* values were not adjusted for multiple comparisons. The box-and-whisker plots depict the minimum and maximum values (whiskers), the upper and lower quartiles, and the median.

We previously reported that HEK293 cells expressing the CD48 S220Y mutant showed markedly reduced CD48 surface levels compared with cells expressing WT CD48, despite similar mRNA levels (14). Accordingly, both patients had lower surface levels of CD48 (Figure 1E). Given that serum levels of soluble CD48 were not previously found to be elevated in P1, and thus secretion was not apparently increased (14), we hypothesized that the S220 CD48 mutant proteins were normally synthesized but had defective cell surface localization. Confocal microscopy of HEK293 cells expressing either the CD48 mutant proteins (S220Y or S220F) or the WT CD48 protein confirmed that the mutant proteins were predominantly localized intracellularly, whereas WT CD48 was primarily detected at the cell surface (Figure 2A).

We further investigated the consequences of S220 CD48 mutant intracellular accumulation, presumably within the ER, where the transamidase complex localizes. To assess ER stress, we generated an ATF4 (activating transcription factor 4) reporter HEK293 cell line and measured reporter fluorescence 24 hours after transfection with S220 CD48 mutants or WT CD48. Whereas cells expressing the S220F mutant had significantly increased ATF4 reporter activity compared with WT cells, indicating enhanced ER stress, a similar trend, although not significant, was observed in cells expressing the S220Y mutant (Figure 2B). Thus, intracellular accumulation of CD48 S220 mutants is associated with activation of ER stress signaling.

We next examined whether the S220 substitutions affect GPI anchoring. The covalent linkage of GPI is initiated within the GPI transamidase by the cysteine protease activity of phosphatidylinositol glycan anchor biosynthesis class K (PIGK), in which the ω-site is positioned for stabilization of the negatively charged intermediate form in an oxyanion hole (22). Substitution with a bulky side group generates steric clashes that alter the positioning of the attacked carbonyl carbon (Figure 1B) but leave the remainder of the C-terminal signal peptide intact (24). We predict that this destabilization of the intermediate form impairs enzyme kinetics. To test this, we treated CD48-transfected HEK293 cells with phosphatidylinositol-specific PLC (PI-PLC), confirming that the identified surface CD48 was indeed GPI anchored (Figure 2, C and D). These mutant proteins are predicted to bind 2B4 and CD2 with binding free energy (reflecting their binding affinities) comparable to WT CD48 (Figure 2E). These findings indicate that the mutant CD48 precursor proteins are able to interact, at least to some extent, with the transamidase complex and that surface-expressed GPI-anchored proteins are likely stable and functional.

Given that a fraction of S220 CD48 proteins undergoes GPI anchoring, we investigated whether coexpression of S220 *CD48* variants affects the surface expression of WT CD48. HEK293 cells were transfected with equal combinations of expression vectors encoding S220 CD48, WT CD48, or empty vectors. Indeed, the combination of WT CD48 with either the S220F or S220Y mutant resulted in significantly lower surface levels of CD48 at 24 and 48 hours compared with the combination of WT CD48 and empty vectors (Figure 2F).

These findings demonstrate that S220 CD48 proteins engage the GPI transamidase but reach the cell surface with reduced efficiency. The CD48 molecules accumulate within the ER, increasing ER stress.

### Activation-associated intracellular CD48 accumulation in patient T cells correlates with impaired survival and function.

**Figure 3:**
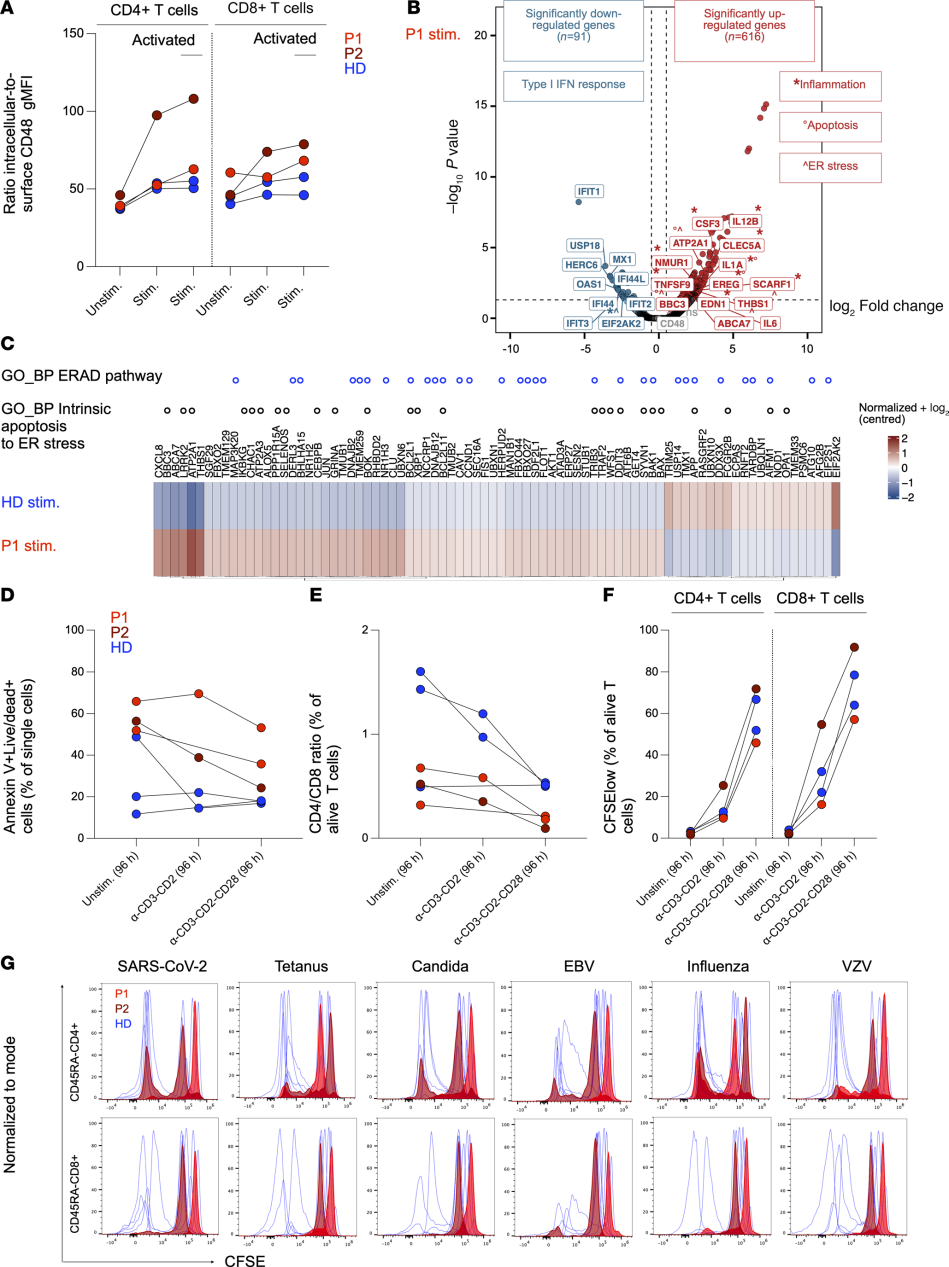
Activation-associated intracellular CD48 accumulation in patient T cells correlates with impaired survival and function. (**A**) Column graph showing the ratio of intracellular/surface CD48 gMFI in unstimulated (unstim.) and stimulated (stim.) CD4^+^ and CD8^+^ T cells, as well as in activated (ICOS^+^CD25^+^) stimulated CD4^+^ and CD8^+^ T cells from P1 (red), P2 (dark red), and 2 HDs (blue). (**B**) Volcano plot showing genes involved in type I IFN response, inflammation (*), apoptosis (°), and ER stress (^) differentially expressed between mitogen-stimulated PBMCs from P1 compared with a HD (log_2_ fold change threshold >1.5; FDR < 0.05). Genes shown in gray are not significantly differentially expressed (ns). (**C**) Heatmap showing expression levels of genes belonging to the BP category “response to ER stress” in mitogen-stimulated PBMCs from a HD compared with P1. Genes that also belong to the BP categories “ERAD pathway” and “intrinsic apoptosis to ER stress” are shown as blue and black circles, respectively. (**D** and **E**) Column graphs showing the fraction of annexin V^+^/Live-Dead^+^ cells and the CD4^+^/CD8^+^ ratio, respectively, in unstimulated and stimulated single cells from P1 (red), P2 (dark red), and 3 HDs (blue). P1 was analyzed twice in 2 independent experiments, and P2 was analyzed once. In one experiment, P1 was compared with 1 HD; in the other, P1 and P2 were compared with 2 HDs. (**F**) Column graphs showing the fraction of CFSE^lo^ cells in unstimulated and stimulated CD4^+^ and CD8^+^ T cells from P1 (red), P2 (dark red), and 2 HDs (blue). (**G**) Overlaid flow cytometry histograms showing T cell divisions by dilution of CFSE dye in memory (CD45RA^–^) CD4^+^ and CD8^+^ T cells from P1 (red), P2 (dark red), and 7 HDs (blue) stimulated with microbial antigens. Due to the low patient sample size in **A** and **D**–**G**, no statistical analysis was performed.

Given the low surface but preserved intracellular CD48 levels previously detected in cells from P1 (14), we hypothesized that CD48 might accumulate intracellularly under conditions of high CD48 synthesis, such as during T cell activation (4). Accordingly, we found that the intracellular/surface CD48 ratio tended to increase upon polyclonal activation using anti-CD3/CD28/CD2 stimulation and was higher in gated activated (ICOS^+^CD25^+^) CD4^+^ and CD8^+^ T cells from both patients compared with healthy donors (HDs) (Figure 3A).

To investigate potential effects of CD48 intracellular accumulation, we compared previously published (14) transcriptomic data from unstimulated and mitogen-stimulated PBMCs derived from P1 to those from a HD. Mitogen-stimulated PBMCs from P1 showed a significant downregulation of genes implicated in type I IFN response to viruses and a significant upregulation of genes involved in inflammation, ER stress, and apoptosis (Figure 3B). This set of genes was not significantly differentially expressed in unstimulated PBMCs (Supplemental Figure 2A). The upregulation of the *IL6* gene was consistent with elevated IL-6 serum levels in both patients (Supplemental Figure 2B). As an initial assessment of the core canonical ER stress response, we examined the expression of genes in the Hallmark Unfolded Protein Response pathway using GSEA but found no significant differences between P1 and HD (Supplemental Figure 2C). We next assessed genes in the broader Biological Process (BP) category response to ER stress from the Gene Ontology database, which includes downstream ER stress–related processes, using GSEA. We observed that 61 of 283 genes in this category were upregulated in P1 (including 21 associated with intrinsic apoptosis), whereas 21 were upregulated in HD (including 4 associated with intrinsic apoptosis) (Figure 3C).

Consistently, PBMCs derived from both patients exhibited reduced viability and increased apoptosis compared with those from HDs, with the differences being more pronounced after anti-CD3/CD2 stimulation (Figure 3D). Among surviving cells, the CD4^+^/CD8^+^ ratio tended to decline following stimulation in patients, indicating that CD4^+^ T cells may be more prone to apoptosis (Figure 3E). Surviving cells remained responsive to polyclonal stimulation (Figure 3F and Supplemental Figure 2D). By contrast, CD3^+^ proliferation and IFN-γ production in response to antigen-specific stimulation tended to be lower in both patients relative to healthy controls (Supplemental Figure 2E), with a more pronounced reduction observed in CD8^+^ T cells (Figure 3G), although prior antigen exposure could be inferred (Supplemental Figure 1A).

Collectively, these findings suggest that S220 *CD48* variants may exert pathogenic effects, particularly during T cell activation, when CD48 is upregulated intracellularly, triggering inflammation, ER stress, and apoptosis and ultimately impairing T cell survival and function.

### TCR repertoire abnormalities in patients with S220 CD48 variants.

**Figure 4:**
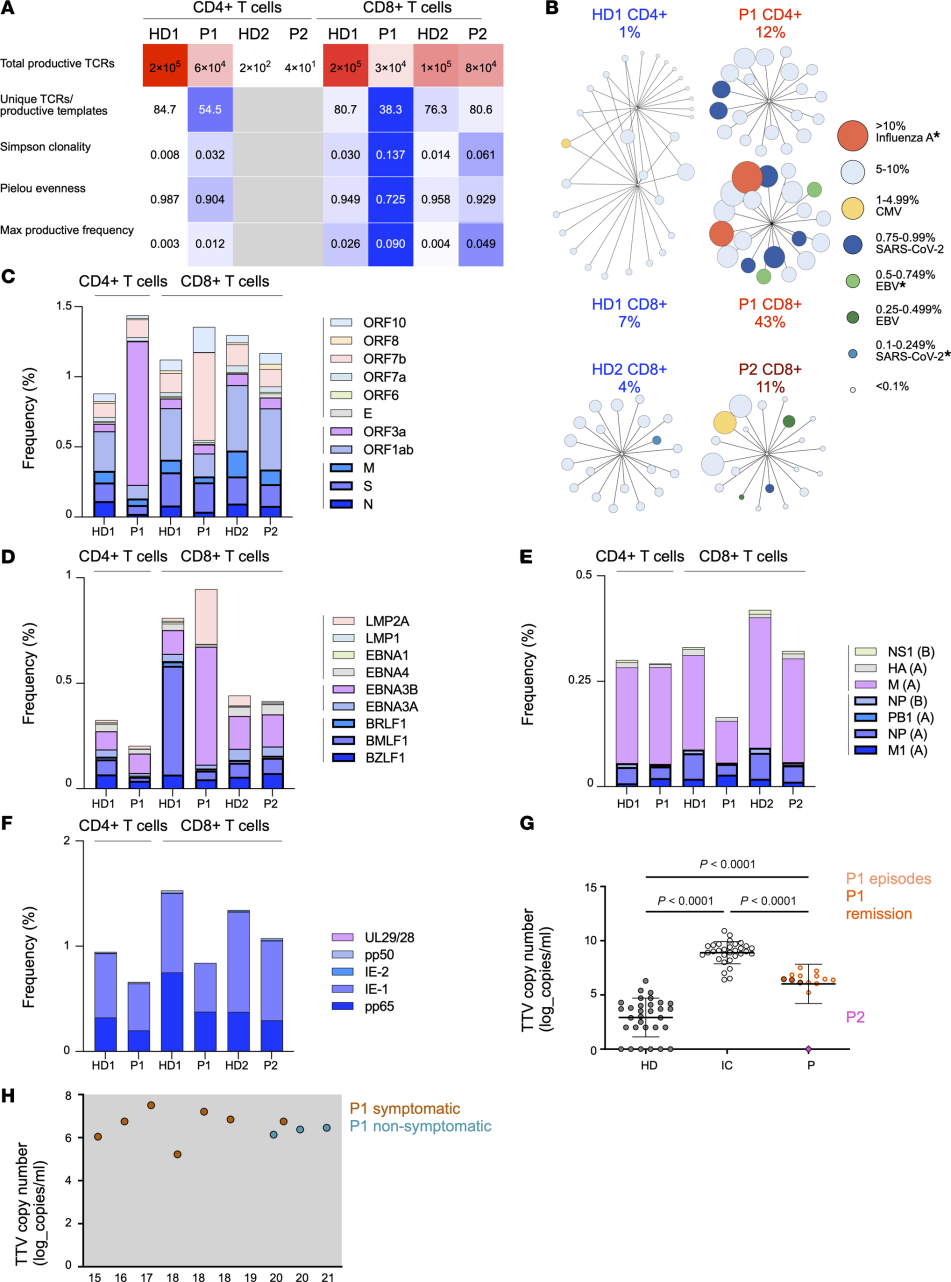
TCR repertoire abnormalities in patients with S220 *CD48* variants. (**A**) Heatmap showing diversity metrics in CD4^+^ and CD8^+^ T cells from P1, P2, and 2 age-matched HDs (HD1 and HD2). The first row has a white-to-red color scale ranging from low (white) to high (red) template numbers. The remaining rows have a blue-to-white color scale ranging from low (blue) to high (white) diversity. (**B**) Cytoscape network representation of the top 20 most abundant clones, showing relative cumulative frequency in CD4^+^ and CD8^+^ T cells from HD1 compared with P1 and CD8^+^ T cells from HD2 compared with P2. Each node represents a clone; node size reflects its relative frequency, and color indicates the predicted antigen specificity. An asterisk indicates multiple predicted specificities, with a representative specificity selected for display. (**C**–**F**) Stacked bar plot illustrating the frequency of putative viral-specific TCRs. Each bar represents a sample, each color indicates a specificity, and the height reflects the cumulative TCR frequency. Thick lines: SARS-CoV-2 structural proteins, EBV lytic antigens, and immunodominant antigens of Influenza A (A) and B (B) viruses; intermediate lines: SARS-CoV-2 nonstructural or accessory immunodominant proteins and EBV latent immunodominant antigens; thin lines: SARS-CoV-2 accessory proteins and EBV less immunodominant antigens. NS1, nonstructural protein 1; M, matrix protein 2; NP, nucleoprotein; PB1, protein PB1-F2; M1, matrix protein 1; UL29/28, protein UL29/28; pp50, DNA polymerase processivity factor; IE-2, 45 kDa immediate-early protein 2; IE-1, immediate early protein IE1; pp65, 65 kDa phosphoprotein. (**G**) Blood TTV copy number (assessed by RT-PCR assay) during inflammatory episodes (light orange) or remission (dark orange) in P1 and P2 (pink) compared with immunocompromised patients (IC; *n* = 31, white) and HDs (*n* = 31, gray) using 1-way ANOVA followed by Tukey’s multiple-comparison test. (**H**) Change in TTV copy number over time in blood from P1 during inflammatory episodes (orange) or remission (light blue).

To assess potential effects of the observed impaired T cell survival and function on immune repertoire dynamics, we performed high-throughput TCR Vβ chain sequencing of magnetically sorted CD4^+^ and CD8^+^ T cells from P1 (age 28 years at assessment), P2 (age 21 years at assessment), and 2 age-matched HDs. While the number of T cells was balanced between patients and HDs, the number of productive (functional and in-frame) complementarity-determining region 3 (CDR3) templates ranged from 26,271 to 182,426 after exclusion of 2 samples with low numbers of templates (Supplemental Table 2). The following parameters were assessed as indicators of repertoire diversity: (a) the number of unique TCRs normalized with the total number of productive templates, (b) the downsampled productive Simpson clonality, (c) the Pielou evenness, and (d) the downsampled maximal productive frequency (Figure 4A). Overall, TCR repertoire diversity was markedly reduced in P1 compared with HD1, with the restriction being more pronounced in CD8^+^ T cells. CD8^+^ T cells from P2 also showed lower diversity, as indicated by the higher Simpson clonality and maximal productive frequency compared with the age-matched HD2 (Figure 4A). Consistently, the top 20 clonotypes (some of which matched viral-specific TCRs included in the Immune Epitope Database [IEDB]) (26) accounted for a larger fraction of the repertoire in CD4^+^ and CD8^+^ T cells from P1 and, less markedly, in CD8^+^ T cells from P2 compared with their respective age-matched controls (Figure 4B and Supplemental Table 3). Moreover, the fraction of overlapping clonotypes between CD4^+^ and CD8^+^ T cells was lower in P1 than in HD1 (Supplemental Figure 3A). A skewed TCR Vβ gene usage was observed in CD8^+^ T cells from P1 and, to a lesser extent, P2 (Supplemental Figure 3B), and CD8^+^ T cells from P1 also exhibited an increased frequency of 42-nucleotide TCRβ CDR3 sequences, suggesting a skewing of the TCR repertoire toward a specific CDR3 length (Supplemental Figure 3C).

To identify TCRs potentially specific for SARS-CoV-2, TCR repertoires were cross-referenced with the ImmuneCODE database (27). P1 showed a reduced cumulative frequency of TCRs predicted to bind the structural proteins membrane (M), spike (S), and nucleocapsid (N) in both CD4^+^ and CD8^+^ T cells, with expansion of putative clones specific for the accessory immunodominant protein ORF3a in CD4^+^ T cells and the accessory proteins ORF7b and ORF10 in CD8^+^ T cells (28) (Figure 4C and Supplemental Table 4). In CD8^+^ T cells from P2, the cumulative frequency of TCRs predicted to bind the structural proteins was slightly reduced, whereas TCRs predicted specific for ORF1ab and ORF3a were largely preserved, and a limited expansion of TCRs potentially specific for the accessory protein ORF8 was observed (Figure 4C and Supplemental Table 4). TCRs predicted to recognize EBV, influenza virus, cytomegalovirus (CMV), and autoantigens were annotated using the VDJdb database (29). In P1, the frequency of TCRs predicted to be specific for lytic, immunodominant EBV peptides (such as BZLF1, BMLF1, and BRLF1) was reduced in both CD4^+^ and CD8^+^ T cells, with expansion of TCRs predicted to bind latent antigens (such as EBNA3B and LMP2A) in CD8^+^ T cells (Figure 4D and Supplemental Table 5). In addition, P1 showed a delayed EBV seroconversion (30, 31) (Supplemental Figure 1A and Supplemental Table 6). P2 exhibited a distribution of putative EBV-specific clonotypes comparable to that observed in HD2, despite the absence of detectable anti-EBV antibodies (Figure 4D and Supplemental Figure 1A). Furthermore, the frequency of putative influenza- and CMV-specific clonotypes was reduced in CD8^+^ T cells from P1 compared with HD1 (Figure 4, E and F). By contrast, the frequency of putative autoantigen-specific clonotypes did not differ between patients and HDs (Supplemental Figure 4D).

Analysis of P1’s plasma samples (collected over 13 years, during both remission and active phases) revealed persistently high levels (compared with controls) of torque teno virus (TTV), a common and typically nonpathogenic virus that has been associated with T cell–directed immunosuppression (32, 33) (Figure 4G). In an RT-PCR assay, the TTV loads during inflammatory episodes and remissions ranged from 10^5^ to 10^7^ copies/mL (Figure 4H). In contrast, the single plasma sample collected from P2 during an asymptomatic period tested negative for TTV (Figure 4G).

Collectively, these data suggest a restriction of TCR repertoire diversity in patients with *CD48* S220 variants, reflecting peripheral clonal T cell expansions.

### Delayed, dysregulated T cell responses to viral infection in CD48^–/–^ mice.

**Figure 5:**
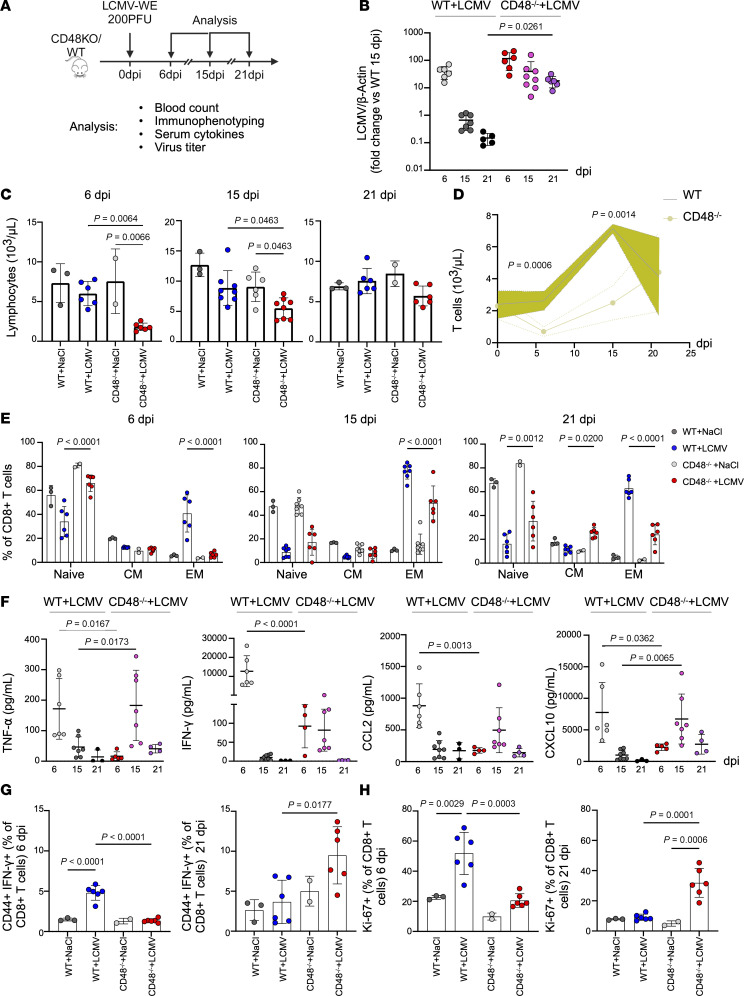
Impaired viral clearance, delayed immune reaction, and inflammation in CD48^–/–^ mice. (**A**) Schematic of the in vivo experiments. 6 days: WT NaCl (*n* = 3), WT LCMV (*n* = 6), CD48^–/–^ NaCl (*n* = 2), CD48^–/–^ LCMV (*n* = 7); 15 days: WT NaCl (*n* = 4), WT LCMV (*n* = 8), CD48^–/–^ NaCl (*n* = 6), CD48^–/–^ LCMV (*n* = 9), 21 days: WT NaCl (*n* = 4), WT LCMV (*n* = 3), CD48^–/–^ NaCl (*n* = 2), WT LCMV (*n* = 6), CD48^–/–^ LCMV (*n* = 6). (**B**) Scatterplot showing real-time PCR analysis of total mRNA isolated from the spleens of WT and CD48^–/–^ mice infected with LCMV at 6, 15, and 21 dpi. LCMV/β-actin relative expression was normalized to the WT at 15 dpi. (**C**) Scatterplots with bars showing blood lymphocyte counts at baseline and postinfection in CD48^–/–^ and WT mice. (**D**) Blood T cell counts during infection in WT and CD48^–/–^ mice. The shaded area represents the SD of the samples. (**E**) Scatterplot with bars showing a comparative analysis of naive, CM, and EM CD8^+^ T cells in the spleen during infection in CD48^–/–^ and WT mice. (**D** and **E**) At each time point, differences between selected groups were assessed using 2-way ANOVA followed by Šidák’s multiple-comparison test. (**F**) Scatterplots with bars of IFN-γ, TNF-α, CCL2, and CXCL10 levels in mouse serum at different time points postinfection. (**G** and **H**) Scatterplots with bars showing the proportions of CD44^+^IFN-γ^+^CD8^+^ T cells in the blood and Ki-67^+^CD8^+^ T cells in the spleen at 6 and 21 dpi. At each time point in **B**, **C**, and **F**–**H**, differences between selected groups were assessed using 1-way ANOVA followed by Tukey’s multiple-comparison test. Graphs in **B**–**H** display the mean ± SD.

Given the above-mentioned important species differences that limit accurate modeling of the human missense variants, we analyzed the progression of Lymphocytic choriomeningitis virus (LCMV) infection in CD48^–/–^mice as a benchmark to evaluate the role of CD48 in antiviral T cell responses and the regulation of inflammation (Figure 5A). This approach was supported by previous data showing that CD48^+/–^ mice also develop an inflammatory phenotype upon LCMV infection (14).

WT mice typically clear LCMV strain WE within 2 weeks (34). However, our results revealed a persistent LCMV WE infection in CD48^–/–^mice up to 21 days postinfection (dpi) (Figure 5B). Following LCMV infection, CD48^–/–^ mice, compared with WT, showed low total circulating lymphocyte counts at 6 and 15 dpi and delayed T cell expansion, as evidenced by elevated counts at 21 dpi only (Figure 5, C and D). Circulating and splenic CD4^+^ and CD8^+^ T cell counts followed a similar temporal pattern, although circulating CD8^+^ T cell numbers remained at the lower range even at 21 dpi in CD48^–/–^ mice (Supplemental Figure 4A). Interestingly, both circulating and splenic CD4^+^ and CD8^+^ EM T cells persisted at the lower range throughout all examined time points in CD48^–/–^ mice (Figure 5E and Supplemental Figure 4A). Signs of delayed response were detectable also in circulating neutrophils and eosinophils, although less marked over time (Supplemental Figure 4B).

Another feature shared with the patients carrying *CD48* S220 variants was an elevated proportion of NK cells that displayed an immature phenotype (Supplemental Figure 1B and Supplemental Figure 4C) and produced more IFN-γ upon stimulation (14) (Supplemental Figure 4D). Multiplex cytokine analysis revealed a delayed immune response in CD48^–/–^ mice, with weak production of key cytokines (TNF-α, IFN-γ, CCL2, and CXCL10) during early time points and then elevated levels at 15 dpi — a time point at which cytokine levels had returned to baseline in WT mice (Figure 5F). The high baseline spleen volume in CD48^–/–^ mice did not change during the early stages of the infection but had risen significantly by 21 dpi and coincided with elevated serum ferritin levels (Supplemental Figure 4E and Supplemental Figure 5A).

We further analyzed T cell responses in LCMV-infected CD48^–/–^ mice. Following LCMV infection, CD48^–/–^ mice showed lower proportions of circulating CD44^+^IFN-γ^+^CD8^+^ T cells and splenic proliferating Ki-67^+^CD8^+^ T cells at early time points compared with WT mice, whereas by 21 dpi these subsets were higher than in WT mice (Figure 5, G and H).

t-SNE and FlowSOM analyses of splenic CD8^+^ T cells at 21 dpi revealed marked differences between WT and CD48^–/–^ mice upon LCMV infection. Specifically, EM cluster 1 was overrepresented in splenic CD8^+^ T cells from WT mice compared with CD48^–/–^ mice, whereas central memory (CM) and EM cluster 4 cells were overrepresented in splenic CD8^+^ T cells from CD48^–/–^ mice compared with WT mice (Supplemental Figure 5B). Further analysis of marker expression indicated that the EM cluster-1 in WT mice corresponded to memory precursor CD8^+^ T cells characterized by the expression of CD127 and CXCR3 and the absence of KLRG1 (35, 36) (Supplemental Figure 5C). In CD48^–/–^ mice, this cluster was underrepresented and relatively enriched in PD-1^+^Ki-67^+^ cells (Supplemental Figure 5C). Conversely, the EM cluster-4 was characterized by the expression of KLRG1, Ki-67, granzyme B, and 2B4 and low expression of CD27, CD127, and PD-1 (Supplemental Figure 5C) and might correspond to terminal effector cells (36). A similar Ki-67^+^2B4^+^granzyme B^+^CD127^lo^ (but KLRG1^lo^PD1^+^CD27^+^, suggesting a less differentiated phenotype) was enriched in the blood of WT mice, whereas it was absent in the blood of CD48^–/–^ mice (Supplemental Figure 5D). These findings suggest that, in CD48^–/–^ mice, persistent viral stimulation may drive late-stage CD8^+^ T cell migration to the spleen and their terminal effector differentiation.

In summary, the absence of CD48 results in impaired viral clearance, inflammation, and delayed, dysregulated immune responses during LCMV infection. These observations emphasize the critical role of CD48 in generating timely and balanced immune responses to viral infections.

## Discussion

In the present study, we reported 2 patients with an inborn error of immunity associated with CD48, as characterized by recurrent inflammatory episodes and impaired T cell survival and responsiveness to antigens. The occurrence of 2 patients with distinct de novo heterozygous S220 variants in *CD48* and similar clinical and immunologic features, in the absence of other known disease-causing *CD48* variants in humans — either monoallelic or biallelic — suggests a disease mechanism specifically attributable to the alteration of the S220 residue (14, 37). Given that S220, which carries a small side chain, is specifically cleaved and covalently attached to the GPI anchor by the transamidase complex (22), amino acid substitutions at this site, particularly with large aromatic amino acids such as tyrosine and phenylalanine, are predicted to impair the efficiency of GPI anchoring (24). We have shown that a small amount of GPI-linked S220Y and S220F CD48 reaches the cell surface. A larger amount of mutant protein is retained intracellularly, suggesting that S220 CD48 mutants engage the GPI transamidase but are modified with reduced efficiency.

Prior work with the GPI-linked prion protein (PrP) demonstrated that in cells with defective GPI synthesis, nascent PrP accumulates and is rapidly removed from the ER by the ER-associated degradation (ERAD) pathways, but only if the C-terminal GPI signal sequence is present (38). Similarly, PrP mutant proteins that were not efficiently cleaved and processed were routed for degradation (38). Additionally, CD48 precursor was identified as a substrate of the ERAD pathway in the ER lumen in an experimental model in which the GPI transamidase was dysfunctional (39). We noted both an accumulation of CD48 and a trend toward increased ATF4/PERK pathway activation in HEK293 cells expressing the S220 mutant proteins, hinting that the mutant proteins with an uncleaved C-terminal signal peptide may remain largely unprocessed and targeted for degradation.

Coexpression experiments raise the possibility that the mutants could also interfere with WT protein processing, possibly through competitive inhibition within the ER or through upregulated degradation pathways, thereby exerting a dominant-negative effect, although a direct mutant–WT protein interaction was not proven.

This proposed model provides a rationale for the unique effects of the altered CD48 cellular distribution, which may involve 3 mechanisms: diminished CD48 surface expression, decreased soluble CD48, and heightened ER stress. The importance of surface CD48 and the consequences of its deficiency are exemplified by CD48^–/–^ mice infected with LCMV, which showed delayed, prolonged inflammation and ineffective viral clearance. The phenotype observed in mice might be more pronounced given the fact that CD48-CD2 interaction is essential for T cell activation in mice, whereas CD2-CD58 provides a similar costimulatory function in human T cells (5). Nevertheless, one would expect that immune features shared between humans and mice may result from the loss of the higher-affinity CD48-2B4 interaction. In fact, similarly to CD48^–/–^ mice, 2B4^–/–^ mice have reduced thymus output of T cells and experience prolonged LCMV infection with ineffective CD8^+^ T cell responses (40). Given that immune-regulatory functions of CD48-2B4 interactions are increasingly recognized as context and cell type specific (12), defects in CD48 expression are expected to have diverse effects on immune regulation. Furthermore, the decreased CD48 binding capacity to bacteria and *C*. *albicans* may directly compromise pathogen clearance (13), and low soluble CD48 may exacerbate inflammation by failing to modulate immune responses effectively. In support of this hypothesis, another study demonstrated that soluble CD48 has a role in dampening basophil activation (41).

Although the mechanistic contribution of ER stress was not extensively examined, our data provide multiple lines of evidence implicating ER stress in the cellular phenotype associated with S220 *CD48* variants. The transcriptomic analysis of mitogen-stimulated PBMCs from P1 revealed upregulation of genes involved in ER stress–associated pathways, inflammation, and apoptosis. Consistent with these findings, expression of S220 *CD48* variants in HEK293 cells resulted in increased ATF4 reporter activity, indicating activation of ER stress signaling.

ER stress is known to be exacerbated under conditions of high protein synthesis (with increased burden of misfolded proteins), such as during T cell activation (42), and might therefore contribute to the enhanced inflammation and increased susceptibility to cell death observed in both patients’ T cells. Apoptosis appeared to be more pronounced in CD4^+^ T cells and was consistent with the lymphopenia seen in both patients and its exacerbation during inflammatory episodes associated with T cell activation. The observation that P1 had predominantly CD4^+^ lymphopenia, whereas P2 showed both CD4^+^ and CD8^+^ lymphopenia, may be explained by a higher degree of CD8^+^ clonal expansion in P1, even though both patients had reduced TCR repertoire diversity. Clonotypic expansions may result from both homeostatic proliferation in the context of lymphopenia and antigen-driven expansion. The presence of a few dominant T cell clonotypes predicted to recognize viral antigens suggests that prolonged antigen stimulation may favor selective expansion of these clones. Although direct evidence of impaired viral control (e.g., delayed EBV seroconversion, early VZV reactivation, and chronic TTV persistence) was obtained only in P1, both patients showed a reduced responsiveness to antigens in vitro. Moreover, the presence of expanded clonotypes predicted to be EBV specific, although in the absence of EBV seroconversion, and the increased CD38 expression on EM T cells may provide indirect evidence of suboptimal viral control in P2 as well.

The T cell abnormalities described in the present study, together with the previously reported defects in NK cell responses (14), are associated with prolonged immune activation and exaggerated inflammation following antigenic challenges, culminating in a clinical phenotype characterized by recurrent inflammatory episodes, during some of which 1 patient fulfilled the diagnostic criteria for HLH (43).

Phenotypic and functional heterogeneity was observed between the patients, with P1 showing a more pronounced phenotype than P2, and might stem from a range of contributing factors, such as age, additional genetic variants, epigenetic influences, or differences in antigen exposure and disease progression. Moreover, the functional consequences of the 2 variants may differ quantitatively, for example, by inducing varying levels of cellular stress.

In conclusion, similarly to approximately one-sixth of all pathogenic missense variants predominantly occurring in proteins that traffic through the secretory pathway (44), S220 *CD48* variants impair CD48 localization to the cell surface and disturb immune cell homeostasis. It is conceivable that the inborn error of immunity associated with CD48 may only become overt in humans if there is a combination of reduced surface expression, low soluble CD48 levels, and intracellular trapping.

Further research, potentially focusing on additional *CD48* variants and on the role of CD48 in immune regulation in multiple contexts, are required to elucidate the precise conditions under which *CD48* variants lead to overt immune dysregulation.

## Methods

Additional details may be found in Supplemental Methods.

### Sex as biological variable.

Given that the pathogenic variants were identified in 2 male patients, male mice were selected for in vivo experiments to match the sex in which the human phenotype manifested. The molecular effects of the variants are expected to be similar in females; however, sex-related differences in immune response could potentially influence the clinical phenotype.

### Sample collection.

Inflammatory episodes were treated with corticosteroids, IVIG, antibiotics, antihistamines, and i.v. fluids (Supplemental Figure 1A). P1 also required occasional thrombocyte transfusions. P1 was treated with cyclosporine between the ages of 14 and 15 years and antibiotic prophylaxis between the ages of 15 and 17 years (Supplemental Figure 1A). Blood samples for PBMC isolation were collected from 2 patients during clinically confirmed remission, at which time they were not receiving any immunosuppressive treatment.

Age-matched HDs were included as controls. PBMCs were isolated using Ficoll-Paque Plus density gradient centrifugation and cryopreserved at –80°C prior to long-term storage in liquid nitrogen.

### Genetic analyses.

Whole-exome sequencing for P1, his brother, and his parents was conducted as described previously (14). Trio WES (Baylor Genetics) was also performed for P2 and P2’s parents.

### Flow cytometry, antibodies, and staining.

Cryopreserved human PBMCs were thawed and automatically counted. Depending on the experiment, approximately 0.2–1 × 10^6^ cells were washed and stained with the Zombie Yellow Fixable Viable Kit (BioLegend) and the following fluorophore-conjugated anti-human antibodies: anti-CD14 BUV395 (MφP9), anti-CD28 BUV496 (CD28.2), anti-IgD BUV563 (IA6-2), anti-CD25 BUV661 (2A3), anti-CD4 BUV737 (RPA-T4), anti-CD31 BUV805 (M89D3), anti-CD3 AmCyan (SK7), anti-CD45RA BV650 (HI100), anti-CD27 FITC (LG.3A10M-T271), anti-CXCR3 PE-CF594 (1C6/CXCR3), anti-CD38 PE-Cy5 (HIT2), anti-CD56 APC (NCAM16.2), anti-CD8 APC-H7 (SK1) (BD Biosciences), anti-CCR7 BV421 (G043H7), anti-CD127 Pacific Blue (A019D5), anti-CD45 BV510 (HI30), anti-CD16 BV570 (3G8), anti-CD39 BV711 (A1), anti–HLA-DR BV750 (L243), anti–PD-1 BV785 (NAT105), anti–Tim-3 PerCP-Cy5.5 (F38-2E2), anti-CCR4 PE (L291H4), anti-CD48 PE-Cy7 (BJ40), anti-CD57 Alexa Fluor 647 (HKN-1), anti-CCR6 Alexa Fluor 700 (G034E3), anti-ICOS Alexa Fluor 647 (C398.4A) (BioLegend), anti-CD19 PerCP-eFluor 710 (HIB19), and anti-CD3 Alexa Fluor 700 (UCHT1) (Thermo Fisher Scientific). To assess T cell proliferation, cryopreserved PBMCs were thawed and labeled at a final concentration of 0.5 μM CFSE (Thermo Fisher Scientific). Apoptosis was assessed using BV711 annexin V staining (BD Biosciences) in annexin V binding buffer (BioLegend).

For the intracellular cytokine analysis, cells were stimulated with 10 ng/mL of phorbol 12-myristate 13-acetate (Sigma-Aldrich) and 1 μg/mL of ionomycin (Sigma-Aldrich) in the presence of 1 μL/mL BD GolgiPlug (BD Biosciences) for 5 hours. After surface staining, the cells were fixed and permeabilized using the fixation/permeabilization solution kit (BD Biosciences). The following fluorophore-conjugated anti-human antibodies were used for intracellular staining: anti–IFN-γ BV421 (B27) (BD Biosciences), anti–IL-4 PE-Cy7 (MP4-25D2), and anti–IL-17A APC/Cy7 (BL168). IL-4 and IL-17A had low staining intensity and therefore are not shown.

The intracellular/surface ratio CD48 was assessed after 2.5 hours incubation with 1 μL/mL Brefeldin A (Sigma-Aldrich). After surface staining with the antibodies described above and anti-CD48 BV421 (Tü145) (BD Biosciences), the cells were fixed and permeabilized, and anti-CD48 PE-Cy7 (BJ40) (BioLegend) was used for intracellular staining.

For mouse spleen and blood samples, red cells were lysed and stained the Zombie Aqua Fixable Viable Kit (BioLegend). Cells were surface stained with the following fluorophore-conjugated anti-mouse antibodies: anti-CD11b BUV395 (M1/70), anti-CXCR3 BUV661 (CXCR3-173), anti-CD44 BUV737 (IM7), anti-CD25 BV480 (PC61), anti-CD48 BUV563 (HM48-1), anti-KLRG1 BUV805 (2F1), anti-CD161 BV605 (PK136), anti-CD95 BV650 (Jo2), anti-CD122 BV750 (5H4), anti-CD127 PerCP-Cy5.5 (A7R34) (BD Biosciences), anti–PD-1 eFluor450 (J43), anti-CD2 NovaFluor Blue 585 (RPA-2.10), anti-CD314 PerCP-eFluor710 (CX5), anti-CD4 PE-Texas Red (RM4-5), anti-CD27 APC (LG.7F9) (Thermo Fisher Scientific), anti-Ly-6C BV570 (HK1.4), anti–Tim-3 BV711 (RTM3-23), anti–I-A/I-E BV785 (M5/114.15.2), anti-CD62L Pacific Blue (MEL-14), anti-CD11c PE-Cy5 (N418), anti-CD244.2 Alexa Fluor 647 [m2B4 (B6)458.1], anti-CD3 Alexa Fluor 700 (17A2), and anti-CD8 APC-Cy7 (BioLegend). The cell suspension was fixed and permeabilized using the eBioscience Foxp3/Transcription Factor Staining Buffer Set (Thermo Fisher Scientific). The following fluorophore-conjugated anti-mouse antibodies were used for intracellular staining: anti–Bcl-6 BUV496 (K112-91), anti–TCF-1/TCF-7 BV421 (S33-966), anti-Perforin (δG9) Alexa Fluor 488 (BD Biosciences), anti–Ki-67 Alexa Fluor 532 (SolA15), anti–IFN-γ PE-Alexa Fluor 610 (XMG1.2), and anti-granzyme B (NGZB) PE-Cy5.5 (Thermo Fisher Scientific).

The samples were acquired on a Cytek Aurora spectral flow cytometer and analyzed using FlowJo software. OmiQ software (Dotmatics) was used to generate t-distributed stochastic neighbor embedding (t-SNE) plots, and FlowSOM software was used to analyze subclusters based on the expression of 29 markers, with the number of clusters chosen arbitrarily. The gating strategies used for flow cytometry are shown in Supplemental Figures 6 and 7.

### Transfection protocol and plasmids.

HEK293 cells (ATCC CRL-1573) were seeded at 2 × 10^6^ cells/10 cm plate in 12 mL culture media with DMEM supplemented with 10% FCS and 1× penicillin/streptomycin and incubated at 37°C with 5% CO_2_ for 48 hours. For transfection, cells were passed at 5 × 10^5^ cells/well in 6-well plates with 2 mL culture media with DMEM supplemented with 10% FCS and incubated at 37°C with 5% CO_2_ for 24 hours to get 50%–70% cell confluency. cDNAs encoding WT CD48 and mutant isoforms were cloned into the pcDNA6.2/EmGFP-Bsd/V5-DEST vector (Thermo Fisher Scientific) without incorporating the V5 tag. Transfection was performed using Lipofectamine 2000 (Thermo Fisher Scientific) with 2.5 μg of plasmid. For cotransfection experiments, 2.5 μg of WT CD48 was combined with 2.5 μg of WT, S220 mutant, or empty vector and then transfected.

To determine whether the surface-localized CD48 was GPI anchored, cells were transfected with WT, S220 mutant, or empty vector and analyzed by flow cytometry following incubation with 1 U/mL PI-PLC from *Bacillus cereus* (Thermo Fisher Scientific) at 37°C for 20 minutes. To measure ATF4-PERK activity, HEK293 cells were transduced with lentiviral particles carrying pLVX-ATF-mScarlet-nuclear localization signal (a gift from David Andrews, Addgene plasmid 115969) (45). Cells were transfected with WT, S220 mutant, or empty vector and analyzed by flow cytometry after 24 hours. mScarlet red fluorescence intensity was quantified as a readout of ATF4 pathway activation.

### Immunofluorescence and microscopy.

48 h after transfection, HEK293 cells lines were fixed in 4% paraformaldehyde for 10 minutes at 37°C. The cells were then blocked in blocking buffer (1% PBS and 5% BSA) for 30 minutes at room temperature. The cells were subsequently incubated with anti-CD48 PE (BJ40; BioLegend) in permeabilization buffer (1% PBS, 2% BSA, and 0.1% Triton-X 100) for 1 hour at room temperature in the dark. The slides were mounted with Prolong Diamond Antifade Mountant (Thermo Fisher Scientific) and acquired with an inverted confocal microscope (Leica DMI6000B, model SP8).

### Serum analyses.

Human cytokines were measured at the Laboratory of Immunology, University Hospital of Zurich, following the standard operating procedures. Murine serum cytokine levels were assayed using the Legendplex Mouse Anti-Virus Response Panel (13-plex) in a V-bottom plate (BioLegend) according to the manufacturer’s instructions. Data were acquired on the Cytek Aurora and analyzed using FlowJo software. The mouse ferritin levels were measured using the mouse ferritin ELISA kit (Crystal Chem) according to the manufacturer’s instructions. The ELISA experiments were analyzed using a BioTek Cytation5 imaging reader.

### T cell stimulation.

Cryopreserved human PBMCs were thawed and counted, and approximately 200,000 cells/well were seeded into a 96 U-bottom plate. The cells were stimulated for 4 days in T cell medium with antibiotin MACSiBead particles loaded with biotinylated anti-CD3/CD28/CD2 antibodies using the T cell Activation/Expansion Kit (Miltenyi Biotec). The T cell medium had the following composition: Iscove’s modified Dulbecco’s medium (Gibco), 5% human serum (Blood Bank Basel), 100 U/mL penicillin-streptomycin (Gibco), and 1% MEM Non-Essential Amino Acids solution (Gibco). The following peptide pools were used for the 10-day antigen stimulation assay: SARS-CoV-2 proteins (N, S, S1, M; PepTivator, Miltenyi Biotec), Tetanus toxin, Candida (MP65), *M*. *tuberculosis* Ag85b, Influenza A (H1N1) HA, VZV (gE, LB01818), EBV (EBNA-1) (peptides & elephants), according to the manufacturer’s instructions. Stimulation with *M*. *tuberculosis* did not elicit a robust response and therefore is not shown.

### RNA-seq.

RNA-seq was performed as previously described (14). The differential expression analysis was performed using the Bioconductor package DESeq2. Genes with an FDR below 0.05 were considered significantly differentially expressed. The *P* values were adjusted for multiple hypothesis testing to control the FDR. Gene sets were obtained from the Molecular Signatures Database.

### Next-generation TCR Vβ chain sequencing.

CD4^+^ and CD8^+^ T cells were isolated from previously frozen PBMCs using the human CD4^+^ and CD8^+^ T cell isolation kit (Miltenyi Biotec). DNA was extracted using the QIAamp DNA Mini Kit (Qiagen) according to the manufacturer’s instructions. TCR Vβ library preparation and sequencing were performed using the immunoSEQ platform at Adaptive Biotechnologies. Productive frequency, diversity metrics, CDR3β length distribution, overlap between CD4^+^ and CD8^+^ T cells, and TCR Vβ gene usage were analyzed using ImmunoSEQ ANALYZER 3.0. Cytoscape was used to visualize networks of the top 20 clones. The specificities of these 20 clones were predicted using the TCRMatch tool (46), which identified TCRs with a match in the IEDB (26) using a high filtering level of >0.97. When multiple specificities were predicted, those with identical scores were all annotated; otherwise, only the specificity with the highest score was annotated. Cumulative productive frequencies of predicted SARS-CoV-2–reactive TCRs were calculated using the ImmunoSEQ T-MAP COVID search tool provided by the ImmunoSEQ Analyzer 3.0 (27). Matches identified based on both rearrangement (exact base–by–base match) and bio-identity (CDR3 amino acid sequence, V gene, and J gene match) were included in the analysis.

ImmunoSEQ TCR sequencing data were exported, converted to a VDJtools-compatible format, and subsequently uploaded to the VDJdb annotation tool for batch querying of the VDJdb database (accessed September 2025) (29). Predicted TCR specificities for EBV, CMV, influenza virus, and autoantigens were annotated, and duplicate TCRs with identical CDR3 sequences were removed either when predicted to recognize the same epitope or retained when recognizing distinct epitopes.

### Mice.

C57BL/6 and B6.129S4-*Cd48^tm1Rsr^*/EpaulJ (common name: slamf2^–/–^) mice were purchased from Jackson Laboratories (strain number 023536) and bred under specific pathogen–free conditions at the University of Zurich.

### LCMV infections.

The LCMV WE strain (200 PFU, provided by Peter Aichele, University of Freiburg Medical Center, Freiburg, Germany) or 0.9% saline solution was injected intraperitoneally into 6- to 8-week-old male mice on day 0. After 6, 15, and 21 days, mice were euthanized for organ and blood collection. Each animal’s body weight was recorded every other day. Part of the spleen was processed in Trizol for real-time PCR measurement of the LCMV, and the rest was used for flow cytometry. A complete blood count was performed on 250 μL of blood at the laboratory of University Animal Hospital, Zurich, Switzerland, 50 μL was used for flow cytometry, and the remainder was used to prepare serum for cytokine assays.

### Statistics.

GraphPad Prism software (version 10) was used for graphing and statistical analysis. Due to the very small sample size, no statistical tests were performed for comparisons of flow cytometry and TCR sequencing data from patients and healthy controls. Computational, serological, and mouse data were compared using 2-tailed *t* tests and 1- or 2-way ANOVA, as specified in the figure legends. The threshold for statistical significance was set to *P* ≤ 0.05.

### Study approval.

Written, informed consent was obtained from all participants, and the study was approved by local investigational review boards in Zurich, Switzerland (reference GSI PB_2016-02280, ClinicalTrials.gov NCT02735824) and Aurora, IL, USA (reference COMIRB_16-0918). All in vivo experiments were approved by the Laboratory Animal Service Center, University of Zurich, and the Zurich Cantonal Veterinary Office (reference ZH158/2020).

### Data availability.

RNA-seq data are deposited in the European Genome-phenome Archive and can be accessed upon request. TCR sequencing data can be accessed via the immuneACCESS database of Adaptive Biotechnologies upon request. All data supporting the findings of this study are included in this article, the supplemental materials, and the Supporting Data Values file.

## Author contributions

SM and TL contributed equally to this work and share first authorship; the author order was determined by the chronology of their involvement in the project. SM confirmed the variants by Sanger sequencing, performed microscopy experiments, conducted initial apoptosis and proliferation assays, and performed in vivo experiments. TL retrieved clinical and immunological data from P1, performed genetic assessments (including structural predictions, population frequency analyses, and evaluation of alternative candidate genes), conducted flow cytometry analyses on patient-derived cells (including immunophenotyping, assessment of surface and intracellular CD48 expression, apoptosis assays, activation and proliferation assays, and antigen stimulation assays), analyzed RNA-seq data, isolated TCRs and analyzed TCR sequencing data; and contributed to writing the revised manuscript. TM and DT helped perform in vivo experiments. RP assessed aspects of T cell biology. OS helped perform microscopy experiments. LM performed molecular dynamics simulations and free-energy calculations. SA, CDW, ZEM, JHR, and ACE performed the transfection experiments. MH performed the viral metagenomics and TTV PCRs on the patient’ plasma samples. SP, CMD, and JPS were responsible for patient care and data collection. SV contributed to assessing aspects of T cell biology. JKA designed and supervised the transfection experiments and contributed to writing the revised manuscript. JPS designed and supervised the project, acquired financial support for the project, wrote the initial manuscript, and contributed to writing the revised manuscript. JKA and JPS contributed equally to this work and share last authorship. All authors reviewed and approved the final manuscript.

## Conflict of interest

The authors have declared that no conflict of interest exists.

## Funding support

- Clinical Research Priority Program CYTIMM-Z of the University of Zurich (to TM and JPS).
- Cancer Research Center of the University of Zurich (to SV and JPS).
- Swiss National Science Foundation (project number 320030_205097; to JPS).
- ITINERARE, a University Research Priority Program of the University of Zurich (to JPS).
- Tara Guetz Foundation and the University of Colorado School of Medicine, Department of Pediatrics (to JKA).
- Clinical Research Priority Program Comprehensive Genomic Pathogen Detection of the University of Zurich (for the metagenomic sequencing).

## Supplementary Material

Supplemental data

Supporting data values

## Version 1 04/15/2026

In-Press Preview

## Version 2 06/01/2026

Electronic publication
