# Supplementary material for: Variants in human CD48 lead to impaired T cell immunity and increased inflammation
Source: J Clin Invest. 2026 Apr 14;136(11):e191340. doi: 10.1172/JCI191340 (PMC13221226; doi:10.1172/JCI191340)
Supplement: Supplemental data [file jci-136-191340-s252.pdf]

Supplemental Material

Variants in human *CD48* Lead to Impaired T-Cell Immunity and Increased Inflammation

Samantha Milanesi[1*], Tiziana Lorenzini[1*], Tommaso Marchetti[1], Diana Tintor[1], Raquel Planas[1,2], Ola Sabet[1,3], Lars Malmström[1], Sudip Acharya[4], Carson D. Williams[4], Zoe E. Manning[4], Jack H. Roser[4], Angelica C. Ehler[4], Michael Huber[5], Seraina Prader[1,6], Stefano Vavassori[1], Cullen M. Dutmer[4], Jordan K. Abbott[4**], and Jana Pachlopnik Schmid[1,6**]

[1]Pediatric Immunology, University of Zurich, Zurich and the Children's Research Center, University Children's Hospital Zurich, Zurich, Switzerland, [2]Department of Cell Biology, Physiology and Immunology, University of Barcelona, Barcelona, Spain, [3]Children's Cancer Hospital Cairo Egypt 57357, Egypt, [4]University of Colorado School of Medicine, Department of Pediatrics, Section of Allergy and Immunology, Aurora, Colorado, [5]Institute of Medical Virology, University of Zurich, Zurich, Switzerland, [6]Division of Pediatric Immunology, University Children's Hospital Zurich, Zurich, Switzerland.

* : co-first authors

**: co-last authors

Corresponding author: Jana Pachlopnik Schmid, Division of Pediatric Immunology, University Children's Hospital Zurich, Lenggstrasse 30, CH-8008 Zurich, Switzerland. +41-44-249-6622. jana.pachlopnik@kispi.uzh.ch

Supplementary Material and Methods

Sanger sequencing *CD48* exon 4

DNA was isolated from blood using the QIAamp DNA Blood Kit (Qiagen). Total genomic DNA was PCR-amplified using GoTaq G2 Hot Start Green Master Mix (Promega) and the following primers: forward 5'-GAGATTGAGGAAAAGAATGTCC-3` and reverse 5'-AGCCTATACAGTCTCTGTCCTG-3'. Sanger sequencing was performed at Microsynth AG using the following primers: forward 5'-GCCCCTTCTCAATACATTAG-3' and reverse 5'-AAAAGAGCTCATCTCAGGTAAG-3'.

Molecular dynamics and free energy calculations

The structure of CD48 was modeled with AlphaFold2 using default parameters (1). Five models each of the CD48-CD2 and CD48-2B4 complexes were generated using AlphaFold2. For each input model, 1000 docking poses were generated using MEGADOCK 4.1.1 (2). Each docking model was subsequently superimposed onto the respective experimentally determined crystal structure using LovoAlign (3). Molecular dynamics (MD) simulations were performed using GROMACS 2024.1 with the AMBER99SB force field (4). Systems were solvated using the SPC water model (spc216 configuration) and neutralized with counterions. After energy minimization and equilibration in canonical (NVT) and isothermal-isobaric (NPT) ensembles, with position restraints, production runs of 100 nanoseconds were carried out with graphics processing units (GPU) acceleration for nonbonded and Particle-Mesh Ewald (PME) interactions. Trajectories were processed with gmx trjconv to remove periodic boundary artifacts and fit trajectories to the protein backbone.

Binding free energies were estimated using the Molecular Mechanics/Poisson–Boltzmann Surface Area (MM/PBSA) method. The calculations were performed using the gmx_MMPBSA tool (version 1.6.4) which interfaces GROMACS trajectories with the

MMPBSA framework (5). Representative snapshots (50 frames) were extracted from the production trajectories. For each frame, the free energy was calculated as the sum of the molecular mechanics gas-phase energy, comprising bonded terms and non-bonded interactions (van der Waals, electrostatic, and 1–4 interactions), and the solvation free energy, comprising polar (Poisson–Boltzmann) and non-polar (solvent-accessible surface area) contributions.

Viral metagenomic sequencing

High-throughput viral metagenomic sequencing was done as previously described (6). Briefly, after virus enrichment and extraction, RNA and DNA were amplified in two separate workflows. A random octamer primer with an anchor sequence was used for reverse transcription (SuperScript III, Thermo Fisher Scientific, Waltham, MA) and one round of second strand synthesis (DNA Polymerase I, Large Klenow Fragment, NEB, Ipswich, MA), respectively. Both workflows were further amplified by the anchor part alone and pooled in equal amounts into library construction with NexteraXT (Illumina, San Diego, CA), prior to sequencing for 150 base pairs on an Illumina MiSeq. Reads were analyzed using an in-house bioinformatics pipeline "VirMet" (6). Reads of viruses identified by the pipeline were used for de novo assembly of contigs using Velvet (7), which were then confirmed by BLAST analysis. Extensive coverage of the whole genome was assured. TTV was confirmed by specific RT-PCR as described (8) .

Supplementary References

1. Jumper J, et al. Highly accurate protein structure prediction with AlphaFold. *Nature.* 2021;596(7873):583-589.
2. Ohue M, et al. MEGADOCK: an all-to-all protein-protein interaction prediction system using tertiary structure data. *Protein Pept Lett.* 2014;21(8):766-778.
3. Martínez L, et al. Convergent algorithms for protein structural alignment. *BMC Bioinformatics.* 2007;8:306.
4. Pronk S, et al. GROMACS 4.5: a high-throughput and highly parallel open source molecular simulation toolkit. *Bioinformatics.* 2013;29(7):845-854.
5. Valdés-Tresanco MS, et al. gmx_MMPBSA: A New Tool to Perform End-State Free Energy Calculations with GROMACS. *J Chem Theory Comput.* 2021;17(10):6281-6291.
6. Lewandowska DW, et al. Unbiased metagenomic sequencing complements specific routine diagnostic methods and increases chances to detect rare viral strains. *Diagn Microbiol Infect Dis.* 2015;83(2):133-138.
7. Zerbino DR, Birney E. Velvet: algorithms for de novo short read assembly using de Bruijn graphs. *Genome Res.* 2008;18(5):821-829.
8. Maggi F, et al. Low prevalence of TT virus in the cerebrospinal fluid of viremic patients with central nervous system disorders. *J Med Virol.* 2001;65(2):418-422.

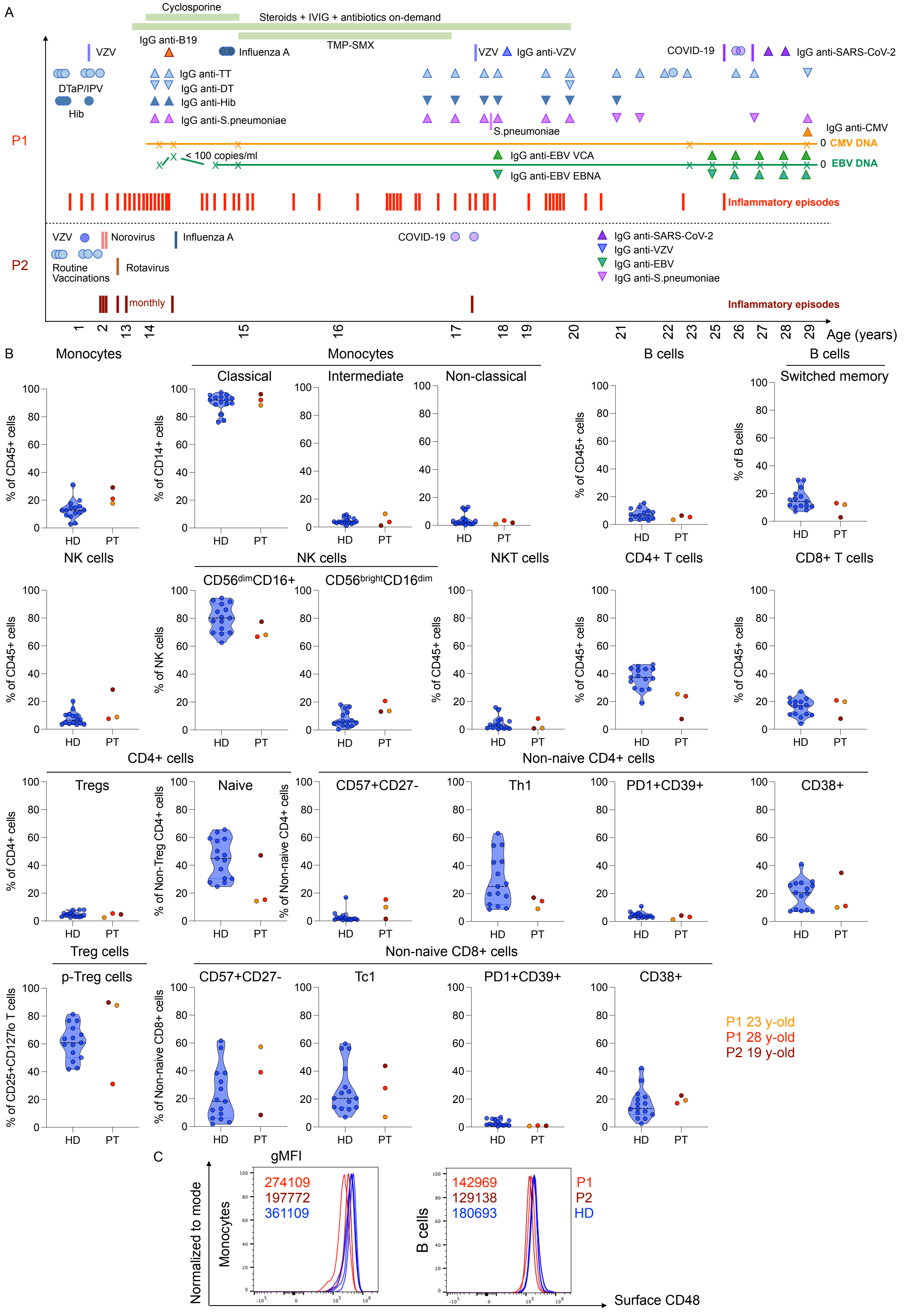
A
Cyclosporine
Steroids + IVIG + antibiotics on-demand
TMP-SMX
IgG anti-B19
VZV
Influenza A
VZV
IgG anti-VZV
COVID-19
IgG anti-SARS-CoV-2
DTaP/IPV
Hib
IgG anti-TT
IgG anti-DT
IgG anti-Hib
IgG anti-S.pneumoniae
S.pneumoniae
IgG anti-CMV
P1
0 CMV DNA
< 100 copies/ml
IgG anti-EBV VCA
IgG anti-EBV EBNA
0 EBV DNA
Inflammatory episodes
VZV
Norovirus
Influenza A
COVID-19
P2
Routine Vaccinations
Rotavirus
monthly
IgG anti-SARS-CoV-2
IgG anti-VZV
IgG anti-EBV
IgG anti-S.pneumoniae
Inflammatory episodes
Age (years)
B
Monocytes
Classical
Intermediate
Non-classical
B cells
Switched memory
NK cells
CD56dimCD16+
CD56brightCD16dim
NKT cells
CD4+ T cells
CD8+ T cells
CD4+ cells
Tregs
Naive
Non-naive CD4+ cells
CD57+CD27-
Th1
PD1+CD39+
CD38+
Treg cells
p-Treg cells
Non-naive CD8+ cells
Tc1
% of CD45+ cells
% of CD14+ cells
% of B cells
% of NK cells
% of CD4+ cells
% of Non-Treg CD4+ cells
% of Non-naive CD4+ cells
% of CD25+CD127lo T cells
% of Non-naive CD8+ cells
HD
PT
P1 23 y-old
P1 28 y-old
P2 19 y-old
C
gMFI
Normalized to mode
Monocytes
274109
197772
361109
B cells
142969
129138
180693
P1
P2
HD
Surface CD48

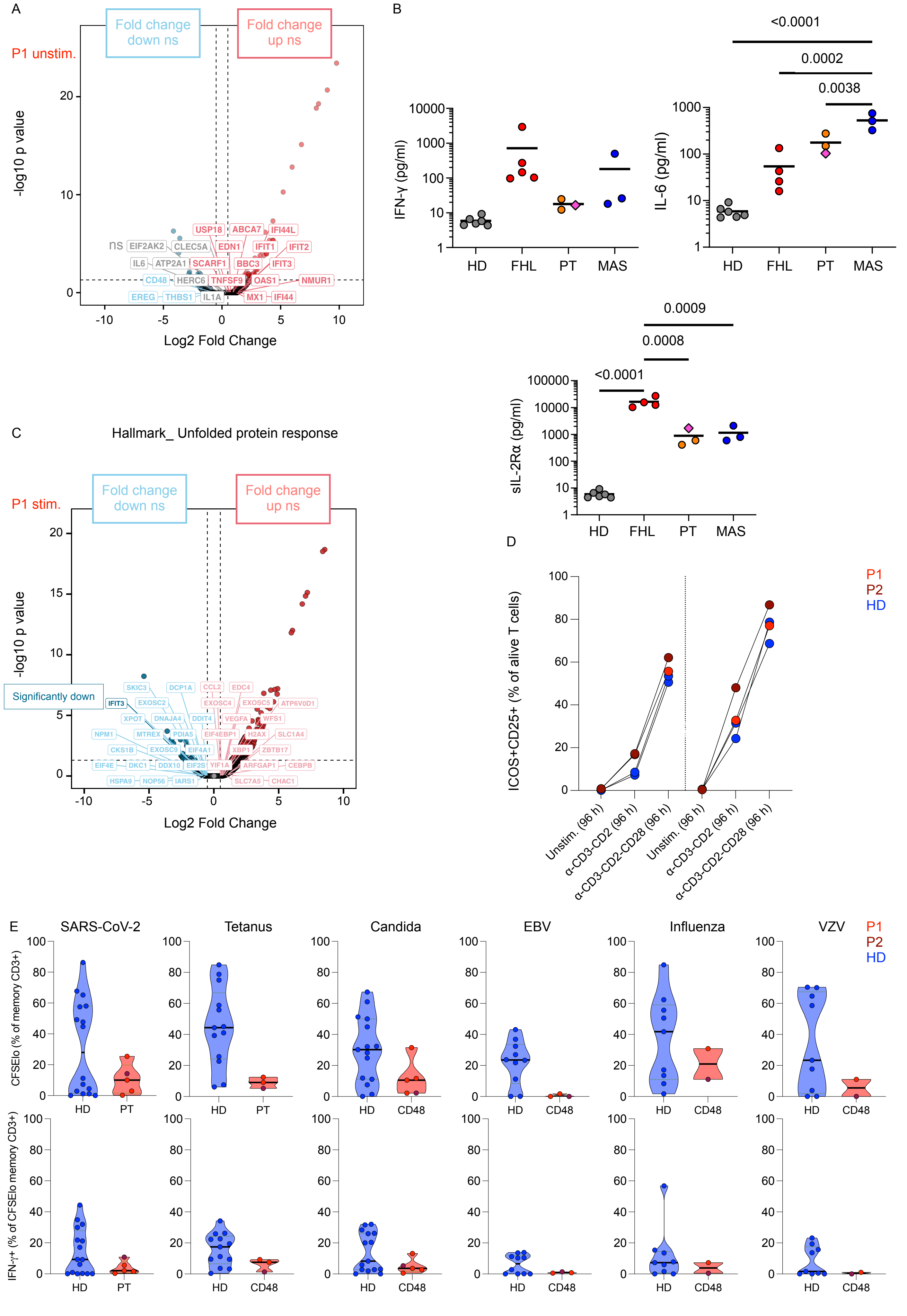

A
P1 unstim.
Fold change down ns
Fold change up ns
-log10 p value
Log2 Fold Change
ns
EIF2AK2
CLEC5A
IL6
ATP2A1
CD48
HERC6
EREG
THBS1
IL1A
USP18
ABCA7
IFI44L
EDN1
IFIT1
IFIT2
SCARF1
BBC3
IFIT3
TNFSF9
OAS1
NMUR1
MX1
IFI44
B
IFN-γ (pg/ml)
IL-6 (pg/ml)
HD
FHL
PT
MAS
<0.0001
0.0002
0.0038
0.0009
0.0008
sIL-2Rα (pg/ml)
C
Hallmark_ Unfolded protein response
P1 stim.
Significantly down
SKIC3
DCP1A
CCL2
EDC4
IFIT3
EXOSC2
EXOSC4
EXOSC5
ATP6V0D1
XPOT
DNAJA4
DDIT4
VEGFA
WFS1
NPM1
MTREX
PDIA5
EIF4EBP1
H2AX
SLC1A4
CKS1B
EXOSC9
EIF4A1
XBP1
ZBTB17
EIF4E
DKC1
DDX10
EIF2S
YIF1A
ARFGAP1
CEBPB
HSPA9
NOP56
IARS1
SLC7A5
CHAC1
D
ICOS+CD25+ (% of alive T cells)
P1
P2
HD
Unstim. (96 h)
α-CD3-CD2 (96 h)
α-CD3-CD2-CD28 (96 h)
E
SARS-CoV-2
Tetanus
Candida
EBV
Influenza
VZV
CFSElo (% of memory CD3+)
IFN-γ+ (% of CFSElo memory CD3+)
HD
PT
CD48

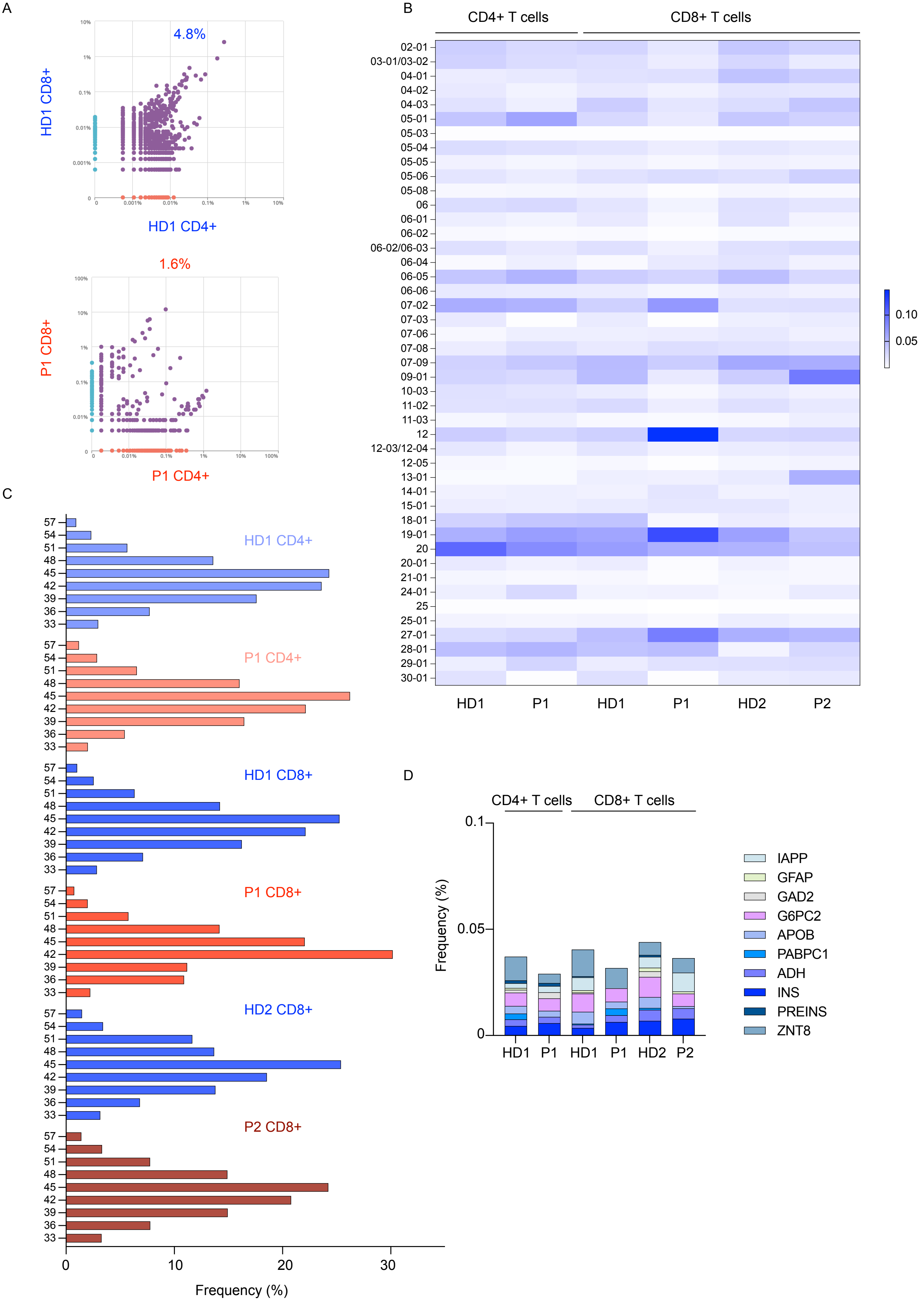
A
HD1 CD8+
4.8%
HD1 CD4+
P1 CD8+
1.6%
P1 CD4+
B
CD4+ T cells
CD8+ T cells
HD1
P1
HD1
P1
HD2
P2
0.10
0.05
C
HD1 CD4+
P1 CD4+
HD1 CD8+
P1 CD8+
HD2 CD8+
P2 CD8+
Frequency (%)
D
CD4+ T cells
CD8+ T cells
Frequency (%)
IAPP
GFAP
GAD2
G6PC2
APOB
PABPC1
ADH
INS
PREINS
ZNT8

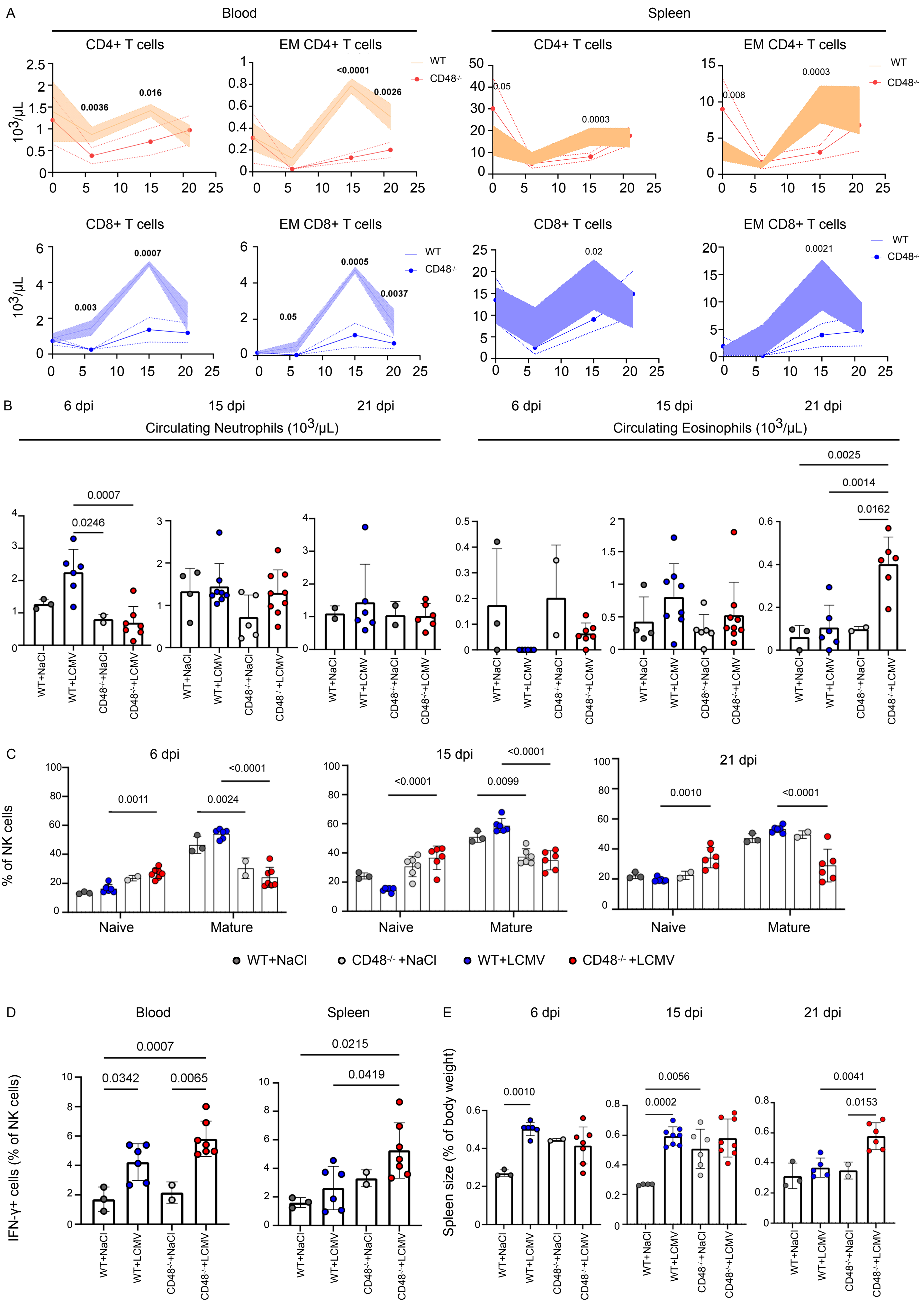
A
Blood
Spleen
CD4+ T cells
EM CD4+ T cells
CD8+ T cells
EM CD8+ T cells
10³/µL
WT
CD48-/-
0.0036
0.016
<0.0001
0.0026
0.05
0.0003
0.008
0.0007
0.0005
0.0037
0.02
0.0021
B
6 dpi
15 dpi
21 dpi
Circulating Neutrophils (10³/µL)
Circulating Eosinophils (10³/µL)
0.0007
0.0246
0.0025
0.0014
0.0162
WT+NaCl
WT+LCMV
CD48-/-+NaCl
CD48-/-+LCMV
C
% of NK cells
0.0011
0.0024
<0.0001
0.0099
0.0010
Naive
Mature
WT+NaCl
CD48-/- +NaCl
WT+LCMV
CD48-/- +LCMV
D
IFN-γ+ cells (% of NK cells)
0.0342
0.0065
0.0215
0.0419
E
Spleen size (% of body weight)
0.0002
0.0056
0.0041
0.0153

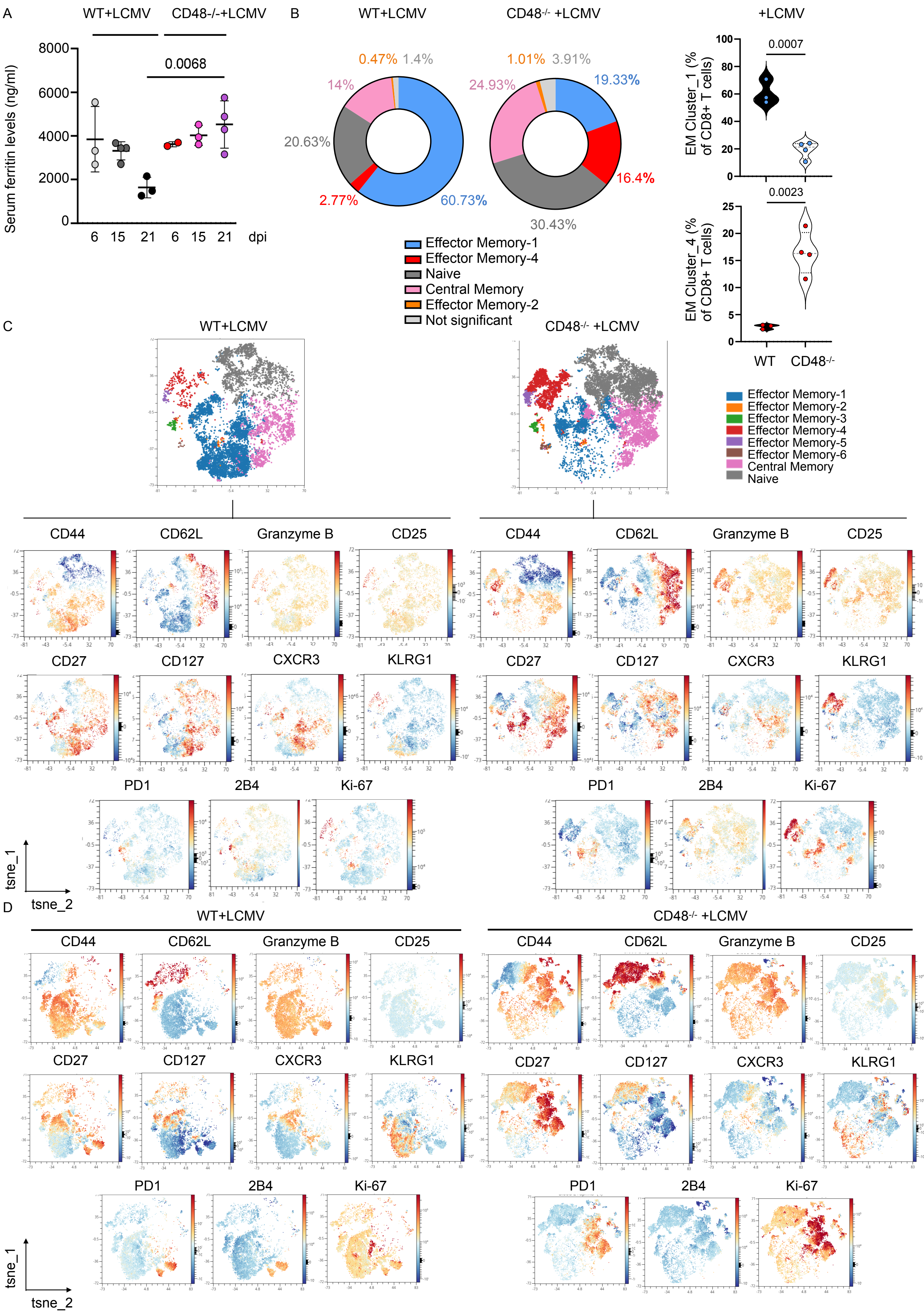
A
WT+LCMV
CD48-/-+LCMV
Serum ferritin levels (ng/ml)
0.0068
dpi
B
WT+LCMV
CD48-/- +LCMV
+LCMV
0.47% 1.4%
14%
20.63%
2.77%
60.73%
1.01% 3.91%
24.93%
19.33%
16.4%
30.43%
Effector Memory-1
Effector Memory-4
Naive
Central Memory
Effector Memory-2
Not significant
EM Cluster_1 (% of CD8+ T cells)
0.0007
EM Cluster_4 (% of CD8+ T cells)
0.0023
WT
CD48-/-
C
WT+LCMV
CD48-/- +LCMV
Effector Memory-1
Effector Memory-2
Effector Memory-3
Effector Memory-4
Effector Memory-5
Effector Memory-6
Central Memory
Naive
CD44
CD62L
Granzyme B
CD25
CD27
CD127
CXCR3
KLRG1
PD1
2B4
Ki-67
tsne_1
tsne_2
D
WT+LCMV
CD48-/- +LCMV
CD44
CD62L
Granzyme B
CD25
CD27
CD127
CXCR3
KLRG1
PD1
2B4
Ki-67
tsne_1
tsne_2

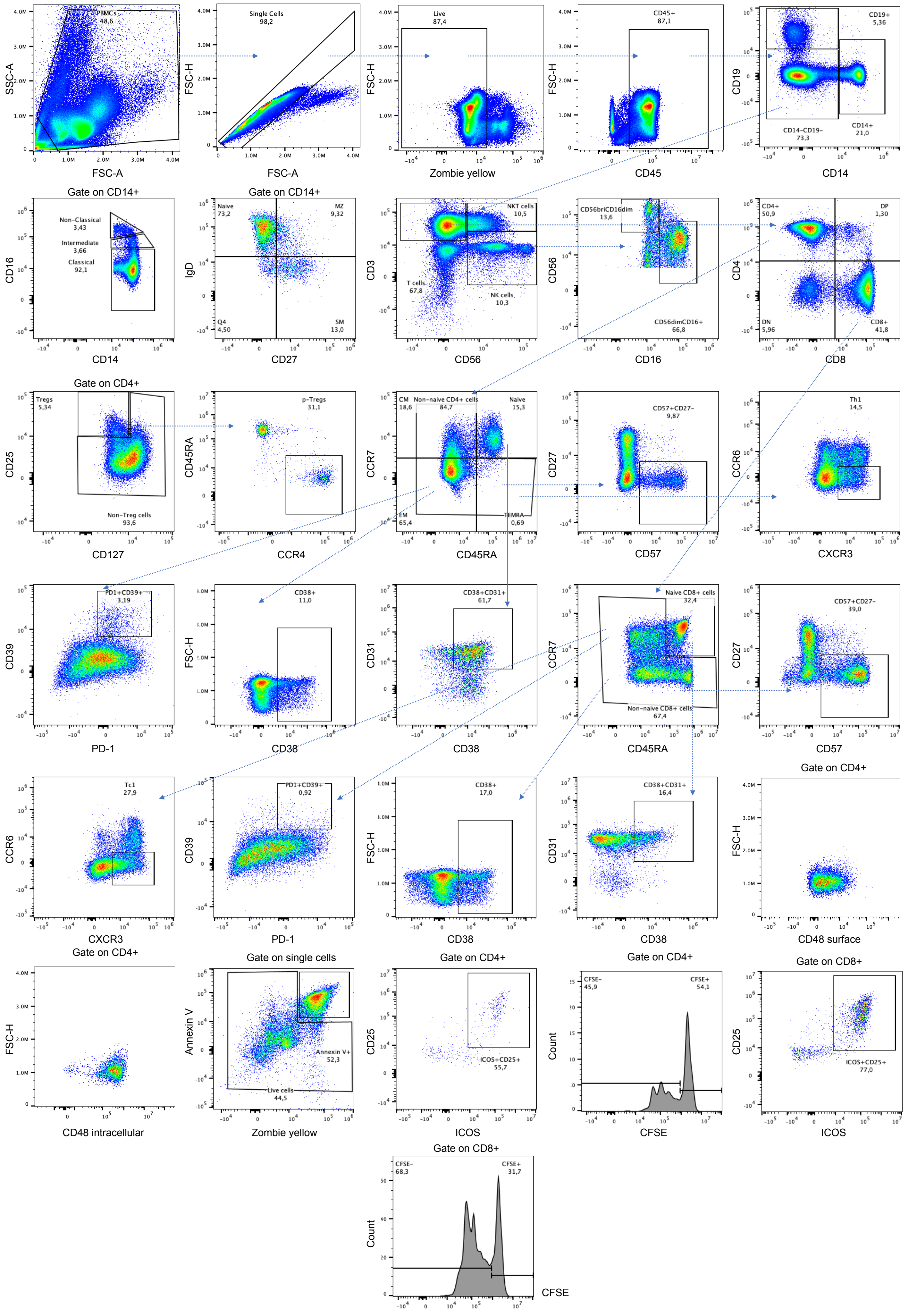
PBMCs 48,6
SSC-A
FSC-A
Single Cells 98,2
FSC-H
Live 87,4
Zombie yellow
CD45+ 87,1
CD45
CD19+ 5,36
CD14-CD19- 73,3
CD14+ 21,0
CD19
CD14
Gate on CD14+
Non-Classical 3,43
Intermediate 3,66
Classical 92,1
CD16
Naive 73,2
MZ 9,32
Q4 4,50
SM 13,0
IgD
CD27
NKT cells 10,5
T cells 67,8
NK cells 10,3
CD3
CD56
CD56briCD16dim 13,6
CD56dimCD16+ 66,8
CD4+ 50,9
DP 1,30
DN 5,96
CD8+ 41,8
CD4
CD8
Gate on CD4+
Tregs 5,34
Non-Treg cells 93,6
CD25
CD127
p-Tregs 31,1
CD45RA
CCR4
CM 18,6
Non-naive CD4+ cells 84,7
Naive 15,3
EM 65,4
TEMRA 0,69
CCR7
CD57+CD27- 9,87
CD57
Th1 14,5
CCR6
CXCR3
PD1+CD39+ 3,19
CD39
PD-1
CD38+ 11,0
CD38
CD38+CD31+ 61,7
CD31
Naive CD8+ cells 32,4
Non-naive CD8+ cells 67,4
CD57+CD27- 39,0
Tc1 27,9
PD1+CD39+ 0,92
CD38+ 17,0
CD38+CD31+ 16,4
CD48 surface
CD48 intracellular
Gate on single cells
Annexin V+ 52,3
Live cells 44,5
Annexin V
ICOS+CD25+ 55,7
ICOS
CFSE- 45,9
CFSE+ 54,1
Count
CFSE
Gate on CD8+
ICOS+CD25+ 77,0
CFSE- 68,3
CFSE+ 31,7

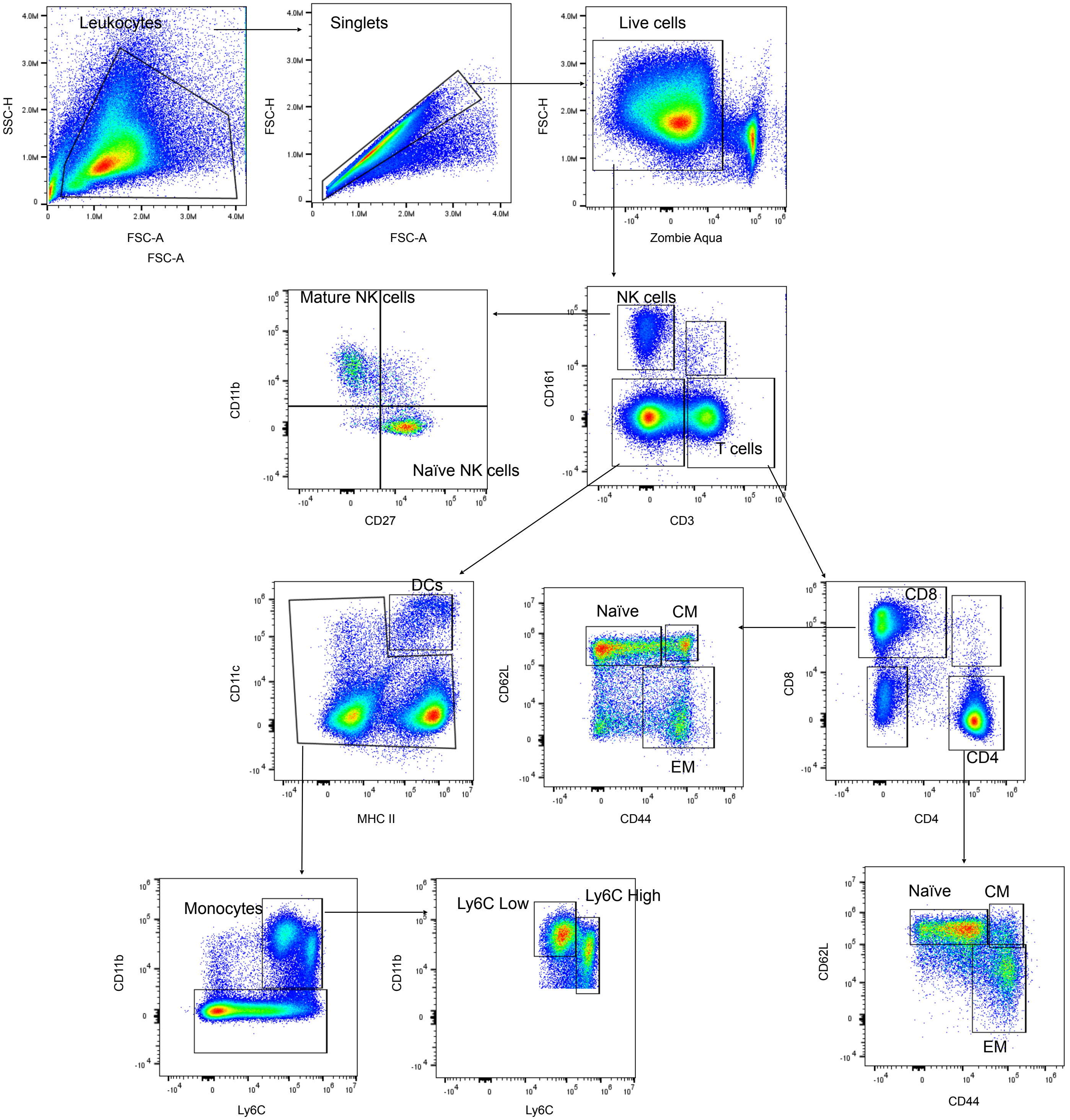
Leukocytes
Singlets
Live cells
SSC-H
FSC-H
FSC-A
Zombie Aqua
Mature NK cells
Naïve NK cells
NK cells
T cells
CD11b
CD27
CD161
CD3
DCs
Naïve
CM
EM
CD8
CD4
CD11c
MHC II
CD62L
CD44
Monocytes
Ly6C Low
Ly6C High
Ly6C

Supplementary Figure Legends

Supplementary Figure 1

A) Longitudinal illustration of the disease course in P1 (red) and P2 (dark red) including timing of vaccinations (dots), infections (vertical bars), inflammatory episodes (red and dark red vertical bars, respectively), and treatments (horizontal bars). Immunoglobulin G levels are indicated by triangles: upward-facing triangles indicate positive serology, downward-facing triangles indicate negative serology. Viral DNA levels for EBV and CMV in P1 are shown. DTaP/IPV: diphtheria (DT), tetanus (TT), acellular pertussis, and inactivated poliovirus combination vaccine. Hib: Haemophilus influenzae type b vaccine. TMP-SMX: trimethoprim-sulfamethoxazole. B19: Parvovirus B19. COVID-19: coronavirus disease 2019.

B) Box and violin plots showing frequencies of blood cell subsets in 15 adult healthy controls (HD, blue dots) as compared to P1 (orange and red dots) and P2 (dark red dots). Values measured at 23 (orange) and 28 years old (red), respectively, are reported for P1, whereas values measured at 19 years old (dark red) are reported for P2. Due to the low patient sample size, no statistical analysis was performed. Truncated violin plots show the median and the quartiles. NKT: natural killer T cells; Tregs: T regulatory cells; Th1: T helper 1; p-Tregs: peripheral Tregs; Tc1: T cytotoxic 1.

C) Overlaid flow cytometry histograms illustrating CD48 surface expression, quantified as geometric mean fluorescence intensity (gMFI), in monocytes and B cells in two adult healthy controls (blue) as compared to P1 (red) and P2 (dark red). Values of gMFI are reported.

Supplementary Figure 2

A) Volcano plot showing the expression, in unstimulated PBMCs from P1 compared with HD, of genes that were significantly differentially expressed in mitogen-stimulated PBMCs.

B) Scatter plot showing serum levels of IFN-γ, IL-6, and soluble IL-2 receptor α (sIL-2Rα) in HDs (grey), patients with familial HLH (FHL, red), P1 (orange dots), P2 (pink dots), and patients with macrophage activation syndrome (MAS) or secondary HLH (blue dots). Data for control subjects and P1 were published previously, whereas the data for P2 are new. One-way ANOVA was performed followed by Tukey's multiple comparisons test.

C) Volcano plot showing genes of the Hallmark Unfolded Protein Response pathway differentially expressed between mitogen-stimulated PBMCs from P1 compared to a HD.

A, C) Log2 fold change threshold >1.5; false discovery rate < 0.05. Grey genes: not differentially expressed (ns: not significant); light red genes: not significantly upregulated; light blue genes: not significantly downregulated, the gene shown in dark blue is significantly downregulated.

D) Column graphs showing the fraction of $ICOS^+CD25^+$ T cells in unstimulated, anti-CD3/CD2, and anti-CD3/CD2/CD28-stimulated $CD4^+$ and $CD8^+$ T cells from P1 (red), P2 (dark red), and two healthy donors (blue).

E) Box and violin plots showing frequencies of $CFSE^{low}$ and IFN-γ^+ $CFSE^{low}$ memory T cells from P1 (red), P2 (dark red), and 13 HD (blue) stimulated with SARS-CoV-2, Tetanus, Candida, EBV, Influenza virus, and VZV antigens. Truncated violin plots show the median and the quartiles. Values from 1-4 experiments are reported for P1.

D-E) Due to the low patient sample size, no statistical analysis was performed.

Supplementary Figure 3

A) Pair-wise scatter plot showing clones that overlap between $CD4^+$ and $CD8^+$ T cells from HD1 (blue) in comparison with P1 (red) and their relative abundance in each sample. The frequency of overlapping clones is indicated.

B) Heat map showing the frequency of $CD4^+$ and $CD8^+$ T cells with a certain TCRβ V gene usage. The TCRβ V gene usage was compared between HD1, P1, HD2, and P2.

C) Spectratyping plot showing frequencies of TCRs with CDR3β length of defined number of nucleotides in $CD4^+$ T cells from HD1 (light blue) and P1 (light red), and $CD8^+$ T cells from HD1 (blue), P1 (red), HD2 (blue), and P2 (dark red).

D) Stacked bar plot illustrating the frequency distribution of clonotypes predicted to recognize autoantigens using the VDJ database. Each bar corresponds to an individual sample, and each color represents a different autoantigen, with height proportional to the cumulative frequency of clonotypes for each specificity. IAPP: islet amyloid polypeptide; GFAP: glial fibrillary acidic protein; GAD2: glutamate decarboxylase 2; G6PC2: glucose-6-phosphatase 2; APOB: apolipoprotein B-100; PABPC1: polyadenylate-binding protein 1; ADH: antidiuretic hormone; INS: insulin; PREINS: preproinsulin; ZNT8: proton-coupled zinc antiporter SLC30A8.

Supplementary Figure 4

A) Counts of $CD4^+$, EM $CD4^+$ (orange), $CD8^+$, and EM $CD8^+$ (blue) cells in the blood and spleen of WT and $CD48^{-/-}$ mice during infection. The shaded area represents the SD of the samples. At each time point, differences between selected groups were assessed using two-way ANOVA followed by Šidák's multiple comparisons test.

B) Scatter plot with bars showing a comparative analysis of circulating neutrophils and eosinophils at baseline and post-infection in $CD48^{-/-}$ and WT mice.

C) Scatter plot with bars showing a comparative analysis of the frequency of splenic NK cells during infection in $CD48^{-/-}$ and WT mice.

D) Scatter plot with bars showing a comparative analysis of the frequency of IFN-γ^+ NK cells in the spleen and blood of mice at 6 dpi and control mice injected with NaCl.

E) Scatter plot with bars showing the spleen-to-body weight ratio at baseline and during infection.

B-E) At each time point, differences between selected groups were assessed using one-way ANOVA followed by Tukey's multiple comparisons test. Graphs display the mean $\pm$ standard deviation (SD).

Supplementary Figure 5

A) Scatter plot with bars showing ferritin concentrations, measured by ELISA, in mouse serum at various time points during infection. One-way ANOVA followed by Tukey's multiple comparisons test was performed.

B) Donut chart representing frequencies of $CD8^+$ T cell subsets assigned using FlowSOM software. On the right, violin plots show the frequency of EM cluster 1 and 4 $CD8^+$ T cells in WT and $CD48^{-/-}$ mice, with the median and quartiles indicated. A two-tailed unpaired t test was performed.

C) T-SNE plot of high-dimensional spectral flow cytometry data gated on $CD8^+$ T cells from the spleens of infected mice at 21 dpi.

D) T-SNE plot of high-dimensional spectral flow cytometry data gated on $CD8^+$ T cells from the blood of infected mice at 21 dpi.

Supplementary Figure 6

Gating strategy used for flow cytometry analysis of human cell subsets. MZ: marginal zone.

Supplementary Figure 7

Gating strategy used for flow cytometry analysis of mouse cell subsets in spleen and blood. DCs: dendritic cells.

Supplementary Tables

Supplementary Table 1. Summary of rare and potentially relevant variants identified in P1 and P2.

P1

Gene	Transcript Consequence	HGVS Consequence	Allele frequency (GnomAD)	Zygosity	Inheritance	REVEL	Constraint	ACMG/ ACGS Classification
STX11	c.616G>A	p.(Glu206Lys)	0.001026	Het	Paternal	0.411	Z=5.58	Likely benign (-3)

P2

Gene	Transcript Consequence	HGVS Consequence	Allele frequency (GnomAD)	Zygosity	Inheritance	REVEL	Constraint	ACMG/ ACGS Classification
PCOLCE2	c.142G>A	p.(Gly48Ser)	0.0	Het	De novo	0.25	NA	VUS (1)
RBMXL2	c.1052C>G	p.(Ser351Cys)	0.0001035	Hom	Both parents (heterozygous)	0.19	NA	VUS (0)
PGK1	c.37G>A	p.(Asp13Asn)	0.000005474	X-linked	Maternal	0.38	NA	VUS (0)

HGVS: Human Genome Variation Society; Het: heterozygous; Hom: homozygous; REVEL: rare exome variant ensemble learner; NA: not available; VUS: variant of uncertain significance.

Supplementary Table 2. Sample characteristics and diversity metrics in CD4+ and CD8+ T cells from patients (P1 and P2) and age-matched healthy controls (HD1 and HD2).

Sample	No. of sorted cells	Amount DNA obtained (ng)	Total productive templates	Unique TCRs	Fraction productive templates (%)	iChao1	Clone distribution slope
HD1_CD4+	1,000,000	5060	182,426	154.565	84.7	1,151.819.2	3.27
P1_CD4+	500,000	972	55,858	30,435	54.5	161,763.7	2.54
HD2_CD4+	200,000	150	205	201	98.1	5,028.5	5.62
P2_CD4+	600,000	135	44	41	93.2	276.2	3.66
HD1_CD8+	500,000	2970	162,269	130,952	80.7	1.722,352.2	2.55
P1_CD8+	400,000	505	26,271	10,059	38.3	113,293.6	2.51
HD2_CD8+	400,000	1220	96,097	73,291	76.3	721,185.7	2.49
P2_CD8+	800,000	2340	84,446	68,040	80.6	834,095	2.85

Supplementary Table 3. Top most expanded clones in each patient, including sample occurrence, amino acid sequence, productive frequency, and IEDB-predicted antigen-specificity.

Sample	Present in	Amino acid	Productive frequency	Score	Source organism
HD1_CD4+	2	CASSPDTGVGYEQYF	0.002751800730159080		
HD1_CD4+	2	CASSPVIGAPGTSGGYTTDTQYF	0.0017705809478912000		
HD1_CD4+	2	CSASIREGNQPQHF	8.66104612281144E-04		
HD1_CD4+	2	CASSLGSGTSGREQYF	6.57800971352768E-04		
HD1_CD4+	1	CASTRGVRDQPQHF	5.97502548978764E-04		
HD1_CD4+	1	CASSQGQFTPYEQYF	5.15277427559668E-04		
HD1_CD4+	2	CASSLDRGVGNTIYF	4.8238737899203E-04		
HD1_CD4+	1	CASSLVKSGNTIYF	4.38533980901845E-04		
HD1_CD4+	1	CSARGDRRNQPQHF	3.67272209005295E-04		
HD1_CD4+	2	CASSEWTGYQPQHF	3.23418810915111E-04	1.000000	CMV
HD1_CD4+	1	CASSTTDSGNTIYF	3.12455461392565E-04		
HD1_CD4+	1	CASSPLGRDTQYF	2.85047087586199E-04		
HD1_CD4+	2	CASYYYGYTF	2.74083738063653E-04		
HD1_CD4+	2	CASSPGRTQGSNSPLHF	2.6860206330238E-04		
HD1_CD4+	2	CASSYSRDRDTYEQYF	2.57638713779834E-04		
HD1_CD4+	2	CASSVVRDRASYEQYF	2.52157039018561E-04		
HD1_CD4+	1	CASSLGRTVLPETQYF	2.46675364257288E-04		
HD1_CD4+	1	CSGTRDYNLYNEQFF	2.41193689496015E-04		
HD1_CD4+	2	CASSWTVVREAFF	2.35712014734742E-04		
HD1_CD4+	1	CASSLQSGGGYEQYF	2.19266990450923E-04		
P1_CD4+	1	CATSLRDRGYNEQFF	0.011887285617100500		
P1_CD4+	1	CASSLETSVYNEQFF	0.009900103834723760	1.000000	SARS-CoV-2
P1_CD4+	1	CASSAEQSSGANVLTF	0.009345125138744680		
P1_CD4+	1	CASSVATLGYGYTF	0.00809194743814673		
P1_CD4+	1	CASSLAAGGGEKLFF	0.007733896666547320		
P1_CD4+	1	CASSSTGDSYNEQFF	0.00753696874216764	0.978925	SARS-CoV-2
P1_CD4+	1	CASSPGRNEKLFF	0.007375845894947900		

P1_CD4+	1	CSARDPRTLDTQYF	0.007196820509148200		
P1_CD4+	1	CSVVHSDFYGYTF	0.006212180887249810		
P1_CD4+	1	CASSRSSGGLQETQYF	0.005549786959790900		
P1_CD4+	1	CVSGRDRGGYEQYF	0.004654660030792370		
P1_CD4+	1	CASSPYSGGAVYTQYF	0.003956461026173510		
P1_CD4+	1	CASSPAGLNTEAFF	0.003938558487593540		
P1_CD4+	1	CASISQGETEAFF	0.003777435640373810		
P1_CD4+	1	CSALNPGGNTIYF	0.003670020408893980		
P1_CD4+	1	CASSLRGAQLQETQYF	0.003652117870314010 0		
P1_CD4+	1	CSAPRTGAPEAFF	0.003526800100254220		
P1_CD4+	1	CSVVHSRNTEAFF	0.003490995023094280		
P1_CD4+	1	CASSLGEGTGANVLTF	0.00334777471445451		
P1_CD4+	1	CASNVQGSTEAFF	0.002864406172795300 0		
HD1_CD8+	2	CASSPDTGVGYEQYF	0.025599467550795300		
HD1_CD8+	2	CASSPVIGAPGTSGGYTTDTQYF	0.008794039526958320		
HD1_CD8+	2	CASSEWTGYQPQHF	0.004720556606622340	1.000000	CMV
HD1_CD8+	2	CASSWTVVREAFF	0.003112116300710550 0		
HD1_CD8+	2	CSASIREGNQPQHF	0.003105953694174490		
HD1_CD8+	2	CASSLDRGVGNTIYF	0.002859449432732070 0		
HD1_CD8+	2	CASYYYGYTF	0.002532831286320860 0		
HD1_CD8+	2	CASSLGSGTSGREQYF	0.002206213139909660		
HD1_CD8+	2	CASSPGRTQGSNSPLHF	0.002193887926837540 0		
HD1_CD8+	2	CASSVVRDRASYEQYF	0.002144587074549050 0		
HD1_CD8+	1	CSAHSKETQYF	0.001984359304611480 0		
HD1_CD8+	1	CASSQDFPAATYEQYF	0.001596115092839670 0		
HD1_CD8+	1	CAWSVRGGESNQPQHF	0.001516001207870880		
HD1_CD8+	1	CSALPTNQETQYF	0.001491350781726640 0		
HD1_CD8+	1	CASSSSGAYTEAFF	0.001472862962118460 0		
HD1_CD8+	2	CASSYSRDRDTYEQYF	0.001318797798716950 0		
HD1_CD8+	1	CASSLDPRGGEQYF	0.001287984766036640 0		
HD1_CD8+	1	CASGSPGTEAFF	0.001281822159500580 0		
HD1_CD8+	1	CASSVEGNPTGELFF	0.001152407422243310		
HD1_CD8+	1	CASSFPAERGTDTQYF	0.001004504865377860 0		

P1_CD8+	1	CASSPNYSNQPQHF	0.11906665144075200	1.000000	Influenza A virus/ SARS coronavirus BJ01
P1_CD8+	1	CASSLTDYGYTF	0.061893342468882000	1.000000	Influenza A virus/ SARS coronavirus BJ02
P1_CD8+	1	CASSIQGWSGQPQHF	0.056602337177876700		
P1_CD8+	1	CASRANSGPSTDTQYF	0.03235506832629140		
P1_CD8+	1	CSARDGGGAYEQYF	0.02478017585931260	0.976333	SARS-CoV-2
P1_CD8+	1	CASSAGGPGYGYTF	0.017433672109931100		
P1_CD8+	1	CASSFTSAGEQYF	0.015720756727951000	0.979722	SARS-CoV-2
P1_CD8+	1	CASSHLPGQGDNEQFF	0.014160100491035700		
P1_CD8+	1	CASSFLTGGQEQYV	0.00989684442921853		
P1_CD8+	1	CASSSLYVGAYEQYF	0.009630390925354960		
P1_CD8+	1	CASSLGRYSYTEAFF	0.008640706482433100		
P1_CD8+	1	CSARVGQEFSGANVLTF	0.00856457690990065		
P1_CD8+	1	CASSLDLQQSTNEKLFF	0.007194244604316550		
P1_CD8+	1	CSVEQGGPYEQYF	0.005976171443797340		
P1_CD8+	1	CASSLSSASGELFF	0.005900041871264890	0.981662	EBV/ Yellow fever virus 17D
P1_CD8+	1	CASSIIGDEQFF	0.005900041871264890	0.990058	SARS-CoV-2
P1_CD8+	1	CASSFSYEQYF	0.005671653153667540	1.000000	EBV/ SARS-CoV2
P1_CD8+	1	CASSLDGFSNQPQHF	0.005481329222336420		
P1_CD8+	1	CSGLGEVRYGYTF	0.005214875718472840		
P1_CD8+	1	CASSLIGETQYF	0.00521487571847284	1.000000	SARS-CoV-2
HD2_CD8+	1	CASSYSQDGEAEKLFF	0.004401802345546690		
HD2_CD8+	1	CASRGTGRGYTF	0.003704590153698870		
HD2_CD8+	1	CASSQSAGTGETYEQYF	0.0035380917198247600		
HD2_CD8+	1	CSARGDRGHTF	0.003049002570319570		
HD2_CD8+	1	RASSLTSGGNTIYF	0.0029033164406797300		
HD2_CD8+	1	CASSLGTGKTNEQFF	0.00271600570257136		
HD2_CD8+	1	CASSPVSGGFGDEQFF	0.0019979812064892800		
HD2_CD8+	1	CSARDLAGSSYNEQFF	0.0018314827726151700		

HD2_CD8+	1	CASSIYPSSGNTIYF	0.0017898581641466400		
HD2_CD8+	1	CASNTGTTNYGYTF	0.0016441720345068000		
HD2_CD8+	1	CATSRDQEGSTRQYF	0.0015609228175697500		
HD2_CD8+	1	CASSQESASGGNEQFF	0.0015297043612183500		
HD2_CD8+	1	CASSVEAGPTPYYEQYF	0.001508892056984090		
HD2_CD8+	1	CASSQDAGRSGANVLTF	0.0014568612963984300		
HD2_CD8+	1	CASSQDRGRQGQGYTF	0.0013423936231099800		
HD2_CD8+	1	CASSYAGYSPLHF	0.0012487382540558000		
HD2_CD8+	1	CARDRGSRTEAFF	0.0012487382540558000		
HD2_CD8+	1	CASSLRQTDEKLFF	0.001155082885001610		
HD2_CD8+	1	CASSFGTDTQYF	0.0011134582765330900	1.000000	SARS-CoV-2/ MTB
HD2_CD8+	1	CASSPSPTPGHTEAFF	0.00100939675536177		
P2_CD8+	1	CASSQGVRVGTYYGYTF	0.048918835705658100		
P2_CD8+	1	CASSLSGSEADTQYF	0.031605996731639200		
P2_CD8+	1	CASSLAGQGSTEAFF	0.014541837387206000	0.971205	CMV
P2_CD8+	1	CASSQTAGSYEQYF	0.0029249461194135900	1.000000	EBV
P2_CD8+	1	CASSLGDRDQETQYF	0.001231556260805720		
P2_CD8+	1	CASSQDSGETQYF	0.0011368211638206700		
P2_CD8+	1	CASSQDRTGGYYGYTF	0.0011249792766975300	1.000000	SARS-CoV-2
P2_CD8+	1	CASSIGGTGRGNSPLHF	0.0010894536153281400		
P2_CD8+	1	CASSLGTGTGVSGNTIYF	0.0010302441797124800		
P2_CD8+	1	CASRRETGLDYGYTF	0.0010065604054662200		
P2_CD8+	1	CASSQDMTGETGELFF	9.35509082727424E-04		
P2_CD8+	1	CASSFLTRIFADTQYF	8.28932098619236E-04		
P2_CD8+	1	CASSQDTSSGANVLTF	7.69722663003576E-04		
P2_CD8+	1	CASSSYPGGRLLGDTQYF	6.98671340264785E-04		
P2_CD8+	1	CASSLRSAAMNTEAFF	6.74987566018521E-04		
P2_CD8+	1	CASSFFQGWEQYF	6.39461904649125E-04		
P2_CD8+	1	CASSLAAGTSIEQYF	6.27620017525993E-04		
P2_CD8+	1	CASTSQAREQYF	6.15778130402861E-04		
P2_CD8+	1	CASSDGTGETDTQYF	5.80252469033465E-04		
P2_CD8+	1	CASSFTGAGANVLTF	5.56568694787201E-04	1.000000	EBV

Supplementary Table 4. Top 62 most expanded clones associated with SARS-CoV-2, including sample occurrence, bio identity (CDR3 amino acid sequence, V gene, and J gene), productive frequency, and the SARS-CoV-2 open reading frames to which the rearrangement is likely to bind.

Sample	Bio identity	Productive frequency	Reference orf
P1_CD4+	CASSLETSVYNEQFF+TCRBV07-08+TCRBJ02-01	0.009900103834723760	ORF3a
P1_CD8+	CASSFSYEQYF+TCRBV07-09+TCRBJ02-07	0.005671653153667540	ORF7b
P1_CD8+	CASSPTGYEQYF+TCRBV03-01/03-02+TCRBJ02-07	0.0015606562369152300	ORF10
HD2_CD8+	CASSFGTDTQYF+TCRBV27-01+TCRBJ02-03	8.94929082073322E-04	M
P1_CD8+	CASSLLGETQYF+TCRBV27-01+TCRBJ02-05	8.75490084123178E-04	S
P1_CD4+	CASSFSYEQYF+TCRBV07-09+TCRBJ02-07	3.93855848759354E-04	ORF7b
P1_CD8+	CASSQGVGYTF+TCRBV04-01+TCRBJ01-02	3.80647862662251E-04	S
P1_CD8+	CASSLETSVYNEQFF+TCRBV07-08+TCRBJ02-01	3.42583076396026E-04	ORF3a
HD2_CD8+	CASSLVGDEQYF+TCRBV05-01+TCRBJ02-07	2.28935346576896E-04	ORF1ab
P1_CD4+	CSASGDGYTF+TCRBV20-01+TCRBJ01-02	1.43220308639765E-04	ORF7b
P1_CD4+	CASSLGGYSNQPQHF+TCRBV07-09+TCRBJ01-05	1.43220308639765E-04	ORF7b
P1_CD4+	CATSDFGSTDTQYF+TCRBV24-01+TCRBJ02-03	1.43220308639765E-04	M
HD1_CD8+	CASSQDGGSGANVLTF+TCRBV04-02+TCRBJ02-06	1.35577343793331E-04	S
P1_CD4+	CASSLNRNYGYTF+TCRBV27-01+TCRBJ01-02	1.25317770059794E-04	ORF7a
HD2_CD8+	CASSLGGGQGLMNTEAFF+TCRBV05-01+TCRBJ01-01	1.14467673288448E-04	ORF7b
HD2_CD8+	CASSFGTDTQYF+TCRBV27-01+TCRBJ02-03	1.14467673288448E-04	M
P1_CD8+	CASSWAETQYF+TCRBV12-03/12-04+TCRBJ02-05	1.14194358798675E-04	ORF10
P1_CD8+	CASSSTGNTEAFF+TCRBV05-06+TCRBJ01-01	1.14194358798675E-04	M
HD2_CD8+	CASSLIGDEQFF+TCRBV05-01+TCRBJ02-01	1.04061521171316E-04	ORF1ab
HD2_CD8+	CASSYSGNQPQHF+TCRBV06-05+TCRBJ01-05	1.04061521171316E-04	ORF1ab
HD2_CD8+	CASSPGQGAYEQYF+TCRBV13-01+TCRBJ02-07	9.36553690541848E-05	S
HD2_CD8+	CASSFGTDTQYF+TCRBV27-01+TCRBJ02-03	9.36553690541848E-05	M
HD2_CD8+	CASSLRNEQFF+TCRBV07-08+TCRBJ02-01	9.36553690541848E-05	ORF7b
P1_CD4+	CSARGPTYEQYF+TCRBV20-X+TCRBJ02-07	8.95126928998532E-05	S

P2_CD8+	CSVVGETQYF+TCRBV29-01+TCRBJ02-05	8.28932098619236E-05	ORF8
P1_CD8+	CASSLGGNTEAFF+TCRBV27-01+TCRBJ01-01	7.61295725324502E-05	ORF1ab
P1_CD8+	CASSLIGDTQYF+TCRBV27-01+TCRBJ02-03	7.61295725324502E-05	ORF1ab
P1_CD8+	CASSYSGDYEQYF+TCRBV06-05+TCRBJ02-07	7.61295725324502E-05	S
P1_CD8+	CASSLSGNYGYTF+TCRBV27-01+TCRBJ01-02	7.61295725324502E-05	ORF1ab
P1_CD8+	CASSLRGSSYEQYF+TCRBV28-01+TCRBJ02-07	7.61295725324502E-05	ORF1ab
HD2_CD8+	CASSYGGGTEAFF+TCRBV06-05+TCRBJ01-01	7.28430648199215E-05	ORF7b
HD2_CD8+	CASSYSGNQPQHF+TCRBV06-05+TCRBJ01-05	7.28430648199215E-05	ORF1ab
HD2_CD8+	CASSGGYSNQPQHF+TCRBV19-01+TCRBJ01-05	7.28430648199215E-05	ORF1ab
P1_CD4+	CASSLGTGDYGYTF+TCRBV07-09+TCRBJ01-02	7.16101543198826E-05	ORF1ab
P1_CD4+	CASSYSTSGDNEQFF+TCRBV06-05+TCRBJ02-01	7.16101543198826E-05	ORF3a
P1_CD4+	CASSLGGYSNQPQHF+TCRBV07-09+TCRBJ01-05	7.16101543198826E-05	ORF7b
HD1_CD4+	CAISESSYEQYF+TCRBV10-03+TCRBJ02-07	7.12617718965498E-05	M
P2_CD8+	CSVVGETQYF+TCRBV29-01+TCRBJ02-05	7.10513227387917E-05	ORF8
P2_CD8+	CASSMGSGEQYF+TCRBV05-04+TCRBJ02-07	7.10513227387917E-05	ORF1ab
HD1_CD8+	CSVGQGYEQYF+TCRBV29-01+TCRBJ02-07	6.77886718966654E-05	ORF3a
HD1_CD8+	CASSPRATNEKLFF+TCRBV27-01+TCRBJ01-04	6.16260653606049E-05	ORF1ab
HD1_CD4+	CAISEGLAGGETQYF+TCRBV10-03+TCRBJ02-05	6.02984223740037E-05	N
P2_CD8+	CSVVGETQYF+TCRBV29-01+TCRBJ02-05	5.92094356156597E-05	ORF8
P2_CD8+	CSASGMSSYNEQFF+TCRBV20-X+TCRBJ02-01	5.92094356156597E-05	ORF7b
P2_CD8+	CASSPPVSGELFF+TCRBV18-01+TCRBJ02-02	5.92094356156597E-05	envelope, ORF1ab
HD1_CD8+	CASSIRSAYEQYF+TCRBV19-01+TCRBJ02-07	5.54634588245444E-05	S
P1_CD4+	CASSPGQGYNEQFF+TCRBV18-01+TCRBJ02-01	5.37076157399119E-05	ORF8
P1_CD4+	CASSLEAGSYNEQFF+TCRBV05-01+TCRBJ02-01	5.37076157399119E-05	ORF7b
P1_CD4+	CASSFSYEQYF+TCRBV07-02+TCRBJ02-07	5.37076157399119E-05	M
P1_CD4+	CASSLGGTEAFF+TCRBV12-03/12-04+TCRBJ01-01	5.37076157399119E-05	ORF1ab
P1_CD4+	CASSLGGYSNQPQHF+TCRBV07-09+TCRBJ01-05	5.37076157399119E-05	ORF7b
HD2_CD8+	CASSLYSNQPQHF+TCRBV27-01+TCRBJ01-05	5.20307605856582E-05	ORF7a
HD2_CD8+	CATSDSYGYTF+TCRBV24-01+TCRBJ01-02	5.20307605856582E-05	M
HD2_CD8+	CASSLGSYGYTF+TCRBV11-02+TCRBJ01-02	5.20307605856582E-05	ORF1ab
HD2_CD8+	CATSRDSGGADTQYF+TCRBV15-01+TCRBJ02-03	5.20307605856582E-05	ORF1ab

P2_CD8+_	CASSEAPGNTIYF+TCRBV02-01+TCRBJ01-03	4.73675484925278E-05	ORF1ab
HD1_CD4+	CASSSTGTSGSYEQYF+TCRBV27-01+TCRBJ02-07	4.38533980901845E-05	ORF7b
HD1_CD4+	CASSYPEGNTEAFF+TCRBV06-06+TCRBJ01-01	4.38533980901845E-05	N
HD1_CD8+	CASSISSTGELFF+TCRBV19-01+TCRBJ02-02	4.31382457524234E-05	S
HD2_CD8+	CASSFYSNQPQHF+TCRBV27-01+TCRBJ01-05	4.16246084685266E-05	ORF7b
HD2_CD8+	CASSQGYEQYF+TCRBV04-01+TCRBJ02-07	4.16246084685266E-05	S
HD2_CD8+	CASSLSEKLFF+TCRBV12-03/12-04+TCRBJ01-04	4.16246084685266E-05	ORF10

Supplementary Table 5. Top 50 most expanded clones associated with EBV, Influenza virus, and CMV including sample occurrence, amino acid sequence, productive frequency, and VDJdb-predicted antigen-specificity.

Sample	Amino acid	Productive frequency	Epitope gene	Epitope species
P1_CD8+	CASSFSYEQYF	0.00472925791912652O	*EBNA3B*	EBV
HD1_CD8+	CASSEWTGYQPQHF	0.00387834355238042O	*pp65*	CMV
HD1_CD8+	CASSEWTGYQPQHF	0.00387834355238042O	*BMLF1*	EBV
P1_CD8+	CASSADGMNTEAFF	0.0026026788548213000	*LMP2A*	EBV
P1_CD8+	CASSSVNEAFF	0.00222179902240843	*pp65*	CMV
HD2_CD8+	CASSFGTDTQYF	7.41468797958374E-04	*IE1*	CMV
P1_CD4+	CASSSGQGNQPQHF	4.79553579213532E-04	*M*	InfluenzaA
P1_CD8+	CASSFGGYTDTQYF	4.76099790516092E-04	*IE1*	CMV
HD2_CD8+	CASSLQGGNYGYTF	4.0522131981446E-04	*LMP2A*	EBV
P1_CD4+	CASSFSYEQYF	3.19702386142355E-04	*EBNA3B*	EBV
HD1_CD8+	CASSLNYEQYF	2.93660477856481E-04	*pp65*	CMV
HD1_CD4+	CASSEWTGYQPQHF	2.6417003595399E-04	*pp65*	CMV
HD1_CD4+	CASSEWTGYQPQHF	2.6417003595399E-04	*BMLF1*	EBV
HD1_CD8+	CASSMGYYGYTF	2.63281807733397E-04	*M*	InfluenzaA
HD1_CD8+	CASSIRGSYEQYF	2.48092472671855E-04	*M*	InfluenzaA
P1_CD4+	CASSLTGPNYGYTF	2.47042752928183E-04	*IE1*	CMV
HD2_CD8+	CASSLAGNTEAFF	2.32786715638094E-04	*IE1*	CMV
HD2_CD8+	CASSLGTGPYEQYF	2.32786715638094E-04	*pp65*	CMV
P1_CD4+	CSARNTGELFF	2.32510826285349E-04	*IE1*	CMV
HD1_CD8+	CASSLGLAGGNEQFF	2.22776914235951E-04	*IE1*	CMV
P1_CD8+	CASSRTSATGELFF	2.22179902240843E-04	*EBNA3B*	EBV
HD2_CD8+	CASSYGTSTDTQYF	1.98299794802821E-04	*M*	InfluenzaA
HD1_CD8+	CASSAGTVAPGELFF	1.92398244112867E-04	*BMLF1*	EBV
P1_CD8+	CASSPGTGNSPLHF	1.90439916206437E-04	*BZLF1*	EBV
P1_CD4+	CASSYPGHYGYTF	1.74383119714012E-04	*M*	InfluenzaA

P1_CD4+	CASSLAGSNYGYTF	1.74383119714012E-04	*pp65*	CMV
P1_CD4+	CASSFGGYEQYF	1.74383119714012E-04	*pp65*	CMV
P1_CD4+	CASSFGGYEQYF	1.74383119714012E-04	*IE1*	CMV
P2_CD8+	CASSQGGYGYTF	1.67500886769401E-04	*IE1*	CMV
HD1_CD8+	CASSPNPAYEQYF	1.67082685676963E-04	*BMLF1*	EBV
P1_CD4+	CASSFSTGANTEAFF	1.59851193071177E-04	*M*	InfluenzaA
P1_CD8+	CASSLVGTDYGYTF	1.58699930172031E-04	*IE1*	CMV
HD1_CD8+	CASSLGVNTEAFF	1.46830238928241E-04	*pp65*	CMV
HD1_CD8+	CASSLGVNTEAFF	1.46830238928241E-04	*NP*	InfluenzaA
P1_CD4+	CASSLIGYEQYF	1.45319266428343E-04	*M1*	InfluenzaA
HD1_CD8+	CASSLGHTGELFF	1.4176712724106E-04	*IE1*	CMV
HD2_CD8+	CASSLQGGNYGYTF	1.37947683341093E-04	*LMP2A*	EBV
P1_CD4+	CASSSTGGDGYTF	1.30787339785509E-04	*M*	InfluenzaA
HD2_CD8+	CASSELAGGQETQYF	1.29325953132275E-04	*IE1*	CMV
HD2_CD8+	CASSQDLAGGPDTQYF	1.29325953132275E-04	*M*	InfluenzaA
P1_CD8+	CASSSGYTEAFF	1.26959944137625E-04	*M1*	InfluenzaA
HD2_CD8+	CASSLVGDGYTF	1.20704222923456E-04	*IE1*	CMV
P2_CD8+	CASSQDRGGQETQYF	1.18235920072518E-04	*EBNA3B*	EBV
HD1_CD8+	CASSLGQGNQPQHF	1.16451568805156E-04	*IE1*	CMV
HD2_CD8+	CASSQTSGGYNEQFF	1.0346076250582E-04	*pp65*	CMV
P1_CD4+	CASSPVSGNTIYF	1.0172348649984E-04	*IE1*	CMV
P1_CD4+	CASSLGLAGYTDTQYF	1.0172348649984E-04	*IE1*	CMV
P1_CD4+	CASSPGTGSYEQYF	1.0172348649984E-04	*BZLF1*	EBV
P1_CD4+	CASSPGTGSYEQYF	1.0172348649984E-04	*IE1*	CMV
P2_CD8+	CASSQGTENTEAFF	9.8529933393765E-05	*IE1*	CMV

Supplementary Table 6. Change over time in P1's anti-EBV antibody profile.

**Age (years):**	**14**	**18**	**25**	**26**	**27**	**28**
Anti-EBV-EBNA-IgG	Borderline	Negative	Negative	**Positive**	**Positive**	**Positive**
Anti-EBV-VCA-IgM	Negative	Negative	Negative	Negative	Negative	Negative
Anti-EBV-VCA-IgG	**Positive**	**Positive**	**Positive**	**Positive**	**Positive**	**Positive**
